# Characterization of Regional Influenza Seasonality Patterns in China and Implications for Vaccination Strategies: Spatio-Temporal Modeling of Surveillance Data

**DOI:** 10.1371/journal.pmed.1001552

**Published:** 2013-11-19

**Authors:** Hongjie Yu, Wladimir J. Alonso, Luzhao Feng, Yi Tan, Yuelong Shu, Weizhong Yang, Cécile Viboud

**Affiliations:** 1 Division of Infectious Disease, Key Laboratory of Surveillance and Early-warning on Infectious Disease, Chinese Centre for Disease Control and Prevention, Beijing, China; 2 Fogarty International Center, National Institutes of Health, Bethesda, Maryland, United States of America; 3 National Institute for Viral Disease Control and Prevention, China CDC, Key Laboratory for Medical Virology, National Health and Family Planning Commission, Beijing, China; Imperial College London, United Kingdom

## Abstract

**Background:**

The complexity of influenza seasonal patterns in the inter-tropical zone impedes the establishment of effective routine immunization programs. China is a climatologically and economically diverse country, which has yet to establish a national influenza vaccination program. Here we characterize the diversity of influenza seasonality in China and make recommendations to guide future vaccination programs.

**Methods and Findings:**

We compiled weekly reports of laboratory-confirmed influenza A and B infections from sentinel hospitals in cities representing 30 Chinese provinces, 2005–2011, and data on population demographics, mobility patterns, socio-economic, and climate factors. We applied linear regression models with harmonic terms to estimate influenza seasonal characteristics, including the amplitude of annual and semi-annual periodicities, their ratio, and peak timing. Hierarchical Bayesian modeling and hierarchical clustering were used to identify predictors of influenza seasonal characteristics and define epidemiologically-relevant regions. The annual periodicity of influenza A epidemics increased with latitude (mean amplitude of annual cycle standardized by mean incidence, 140% [95% CI 128%–151%] in the north versus 37% [95% CI 27%–47%] in the south, *p*<0.0001). Epidemics peaked in January–February in Northern China (latitude ≥33°N) and April–June in southernmost regions (latitude <27°N). Provinces at intermediate latitudes experienced dominant semi-annual influenza A periodicity with peaks in January–February and June–August (periodicity ratio >0.6 in provinces located within 27.4°N–31.3°N, slope of latitudinal gradient with latitude −0.016 [95% CI −0.025 to −0.008], *p*<0.001). In contrast, influenza B activity predominated in colder months throughout most of China. Climate factors were the strongest predictors of influenza seasonality, including minimum temperature, hours of sunshine, and maximum rainfall. Our main study limitations include a short surveillance period and sparse influenza sampling in some of the southern provinces.

**Conclusions:**

Regional-specific influenza vaccination strategies would be optimal in China; in particular, annual campaigns should be initiated 4–6 months apart in Northern and Southern China. Influenza surveillance should be strengthened in mid-latitude provinces, given the complexity of seasonal patterns in this region. More broadly, our findings are consistent with the role of climatic factors on influenza transmission dynamics.

*Please see later in the article for the Editors' Summary*

## Abstract 2

Cécile Viboud and colleagues describe epidemiological patterns of influenza incidence across China to support the design of a national vaccination program.

*Please see later in the article for the Editors' Summary*

## Editors' Summary

**Background:**

Every year, millions of people worldwide catch influenza, a viral disease of the airways. Most infected individuals recover quickly but seasonal influenza outbreaks (epidemics) kill about half a million people annually. These epidemics occur because antigenic drift—frequent small changes in the viral proteins to which the immune system responds—means that an immune response produced one year provides only partial protection against influenza the next year. Annual vaccination with a mixture of killed influenza viruses of the major circulating strains boosts this natural immunity and greatly reduces the risk of catching influenza. Consequently, many countries run seasonal influenza vaccination programs. Because the immune response induced by vaccination decays within 4–8 months of vaccination and because of antigenic drift, it is important that these programs are initiated only a few weeks before the onset of local influenza activity. Thus, vaccination starts in early autumn in temperate zones (regions of the world that have a mild climate, part way between a tropical and a polar climate), because seasonal influenza outbreaks occur in the winter months when low humidity and low temperatures favor the transmission of the influenza virus.

**Why Was This Study Done?:**

Unlike temperate regions, seasonal influenza patterns are very diverse in tropical countries, which lie between latitudes 23.5°N and 23.5°S, and in the subtropical countries slightly north and south of these latitudes. In some of these countries, there is year-round influenza activity, in others influenza epidemics occur annually or semi-annually (twice yearly). This complexity, which is perhaps driven by rainfall fluctuations, complicates the establishment of effective routine immunization programs in tropical and subtropical countries. Take China as an example. Before a national influenza vaccination program can be established in this large, climatologically diverse country, public-health experts need a clear picture of influenza seasonality across the country. Here, the researchers use spatio-temporal modeling of influenza surveillance data to characterize the seasonality of influenza A and B (the two types of influenza that usually cause epidemics) in China, to assess the role of putative drivers of seasonality, and to identify broad epidemiological regions (areas with specific patterns of disease) that could be used as a basis to optimize the timing of future Chinese vaccination programs.

**What Did the Researchers Do and Find?:**

The researchers collected together the weekly reports of laboratory-confirmed influenza prepared by the Chinese national sentinel hospital-based surveillance network between 2005 and 2011, data on population size and density, mobility patterns, and socio-economic factors, and daily meteorological data for the cities participating in the surveillance network. They then used various statistical modeling approaches to estimate influenza seasonal characteristics, to assess predictors of influenza seasonal characteristics, and to identify epidemiologically relevant regions. These analyses indicate that, over the study period, northern provinces (latitudes greater than 33°N) experienced winter epidemics of influenza A in January–February, southern provinces (latitudes less than 27°N) experienced peak viral activity in the spring (April–June), and provinces at intermediate latitudes experienced semi-annual epidemic cycles with infection peaks in January–February and June–August. By contrast, influenza B activity predominated in the colder months throughout China. The researchers also report that minimum temperatures, hours of sunshine, and maximum rainfall were the strongest predictors of influenza seasonality.

**What Do These Findings Mean?:**

These findings show that influenza seasonality in China varies between regions and between influenza virus types and suggest that, as in other settings, some of these variations might be associated with specific climatic factors. The accuracy of these findings is limited by the short surveillance period, by sparse surveillance data from some southern and mid-latitude provinces, and by some aspects of the modeling approach used in the study. Further surveillance studies need to be undertaken to confirm influenza seasonality patterns in China. Overall, these findings suggest that, to optimize routine influenza vaccination in China, it will be necessary to stagger the timing of vaccination over three broad geographical regions. More generally, given that there is growing interest in rolling out national influenza immunization programs in low- and middle-income countries, these findings highlight the importance of ensuring that vaccination strategies are optimized by taking into account local disease patterns.

**Additional Information:**

Please access these websites via the online version of this summary at http://dx.doi.org/ 10.1371/journal.pmed.1001552.

- This study is further discussed in a *PLOS Medicine* Perspective by Steven Riley
- The UK National Health Service Choices website provides information for patients about seasonal influenza and about influenza vaccination
- The World Health Organization provides information on seasonal influenza (in several languages) and on influenza surveillance and monitoring
- The US Centers for Disease Control and Prevention also provides information for patients and health professionals on all aspects of seasonal influenza, including information about vaccination; its website contains a short video about personal experiences of influenza.
- Flu.gov, a US government website, provides access to information on seasonal influenza and vaccination
- Information about the Chinese National Influenza Center, which is part of the Chinese Center for Disease Control and Prevention: and which runs influenza surveillance in China, is available (in English and Chinese)
- MedlinePlus has links to further information about influenza and about vaccination (in English and Spanish)
- A recent *PLOS Pathogens* Research Article by James D. Tamerius et al. investigates environmental predictors of seasonal influenza epidemics across temperate and tropical climates
- A study published in *PLOS ONE* by Wyller Alencar de Mello et al. indicates that Brazil, like China, requires staggered timing of vaccination from Northern to Southern states to account for different timings of influenza activity.

## Introduction

The seasonality of influenza has been well studied in temperate regions of the world but remains poorly characterized in tropical and subtropical areas [1],[2]. A growing body of evidence suggests that seasonal patterns are highly diverse in tropical settings, particularly in Asia, where influenza can display semi-annual or annual epidemic cycles, as well as year-round activity [2]–[4]. Experimental and modeling studies have suggested that low levels of absolute humidity and cold temperature favor influenza transmission and survival in temperate settings [5]–[8], while rainfall fluctuations may drive influenza activity in low latitudes [8]. From a public health perspective, local information on influenza seasonality and circulating strains is crucial to inform the timing and composition of influenza vaccines, particularly for large tropical countries [9]. In parallel, there is growing interest in establishing routine immunization programs in low- and middle-income regions [10], due to strengthening of laboratory surveillance systems and increased recognition of disease burden [11]–[14].

China is a geographically, economically, and climatologically diverse country with a population of 1.34 billion, which experiences substantial influenza mortality burden, estimated at 11–18 excess deaths per 100,000 in pandemic and inter-pandemic seasons [11],[15]. Although seasonal influenza vaccination was introduced in 1998, China has yet to initiate a national immunization program [16]. Previous work has suggested intriguing differences in the seasonality and evolutionary dynamics of influenza between Northern and Southern China [4],[17],[18], indicating that analysis of high-resolution epidemiological data will be required to guide control strategies in this country. The goals of our study were to characterize the seasonality of the disease across China, assess the role of putative drivers of seasonality, and identify broad epidemiological regions that could be used as a basis to optimize the timing of future vaccination programs.

## Methods

### Influenza Surveillance Dataset

We used weekly reports from a national sentinel hospital-based influenza surveillance network, providing the number of laboratory-confirmed influenza cases by virus type (influenza A and B) and the number of specimens tested in 30 Chinese provinces. Influenza laboratory surveillance was initiated in 2000 in China; here we focused on the period 2005–2011 where sampling was more intense. We briefly describe the surveillance system below; refer to [11] for more details.

**Figure 1:**
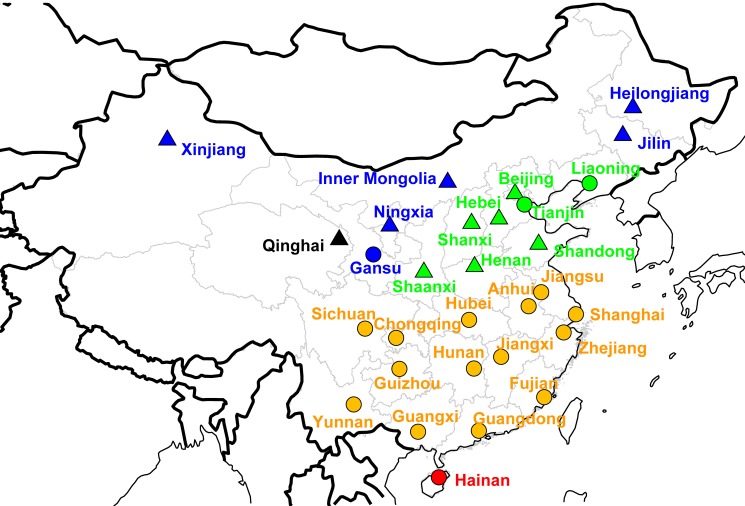
Map of Chinese provinces conducting influenza surveillance (*n* = 30). Dots indicate the location of the capital city in each province. A total of 193 hospitals participate in disease surveillance, representing 88 cities. Colors illustrate different climatic domains (black, cold-temperate; blue, mid-temperate; green, warm-temperate; orange, subtropical; red, tropical). Different symbols indicate the type of surveillance scheme (circles, year-round surveillance; triangles, Oct through Mar surveillance).

Each week, 193 sentinel hospitals located in 88 cities representing all 30 provinces with exception of Tibet (Figure 1) reported the number of patients with influenza-like-illness (ILI) and total visits to outpatient and/or emergency departments to a centralized online system maintained by Chinese Center for Disease Control and Prevention (China CDC, Beijing). Identification of patients with ILI was based on a standard case definition, including body temperature ≥38°C with either cough or sore throat, in the absence of an alternative diagnosis. In each sentinel hospital, nasopharyngeal swabs were collected daily from the first one or two ILI cases and placed in sterile viral transport medium for influenza virus testing, resulting in ten to 15 specimens per hospital per surveillance week. Samples were inoculated into Madin-Darby canine kidney (MDCK) cells and/or specific pathogen free (SPF) chicken embryo for virus isolation. Hemagglutination inhibition (HI) and/or conventional or real-time reverse transcription PCR (RT-PCR) assay were performed to identify the types/subtype of influenza virus, following a standard protocol [19].

Because of limited understanding of the seasonality of influenza in China prior to this study, and following general WHO recommendations, influenza surveillance was implemented year-round in 99 Southern Chinese hospitals, representing 45 cities below latitude 34°N. To confirm the seasonality of influenza in Northern China, surveillance activities were also conducted year-round in 22 of the 94 northern hospitals, while surveillance was restricted to the period October to March in the remaining hospitals (representing 43 cities total; Figure 1). In response to the evolving A/H1N1 pandemic, all 193 sentinel hospitals were asked to implement surveillance year-round starting in May 2009. All 193 participating hospitals have contributed systematic information during the study period, so that the number of participating sites remained constant. Since China is located in the Northern Hemisphere, we defined the respiratory season as the period running from August 1st to July 31st each year. Analysis excluded the A/H1N1 pandemic season April 2009–April 2010 to focus on influenza patterns in inter-pandemic seasons.

### Climate, Geographic, and Demographic Data

To assess the role of putative drivers of influenza seasonality, we collected province-level demographic, economic, and geographic data, including population size and density [20], gross domestic product [20], and human mobility patterns between provinces (including the per capita number of passengers travelling by air, rail, road, and boat [21]). Average latitude and longitude coordinates for each province were obtained by weighting the coordinates of cities participating in influenza surveillance by their population sizes (Table 1).

**Table 1:** Background characteristics of the 30 provinces involved in influenza surveillance and information on influenza sampling intensity, 2005–2011, China.

Province (Climate)	*n* Cities (Hosp)^a^	Population Size (M)	Latitude (°N)	Longitude (°E)	Per Capita GRP (2009) $	Per Capita Annual Passenger Fluxes	Mean Monthly Temperature (Min, Max) °C	Mean Monthly Air Pressure (Min, Max) Bar	Mean Monthly Relative Humidity (Min, Max) %	Mean Monthly Rainfall (Min, Max) mm	Mean Monthly Hours Sunshine (Min, Max)	Mean Annual *n* Specimens Tested (rate per 10,000)	Mean Annual *n* Influenza Positive
Anhui^b^ (ST)	2 (5)	5.9	31.8	117.5	2,402	219.5	17 (3, 29)	1.01 (1, 1.02)	74 (68, 83)	32 (30, 37)	11 (4, 20)	2,273 (38.6)	360
Beijing (WT)	1 (5)	16.1	39.9	116.4	10,313	77.7	13 (−2, 27)	1.01 (1, 1.02)	53 (39, 70)	23 (17, 32)	4 (0, 9)	2,122 (13.2)	485
Chongqing^b^ (ST)	1 (5)	13.8	29.6	106.6	3,355	77.2	19 (8, 29)	0.98 (0.97, 0.99)	79 (73, 86)	34 (32, 38)	12 (3, 22)	1,892 (13.7)	436
Fujian^b^ (ST)	4 (9)	19.9	25.3	118.8	4,954	36.1	21 (11, 29)	1 (0.99, 1.01)	73 (65, 80)	32 (29, 34)	15 (3, 29)	4,231 (21.3)	701
Gansu^b^ (MT)	3 (6)	8.4	35.6	104.7	1,884	54.4	9 (−6, 22)	0.84 (0.83, 0.84)	59 (47, 71)	26 (21, 31)	3 (0, 6)	2,213 (26.2)	389
Guangdong^b^ (ST)	3 (9)	21.1	22.9	113.4	6,026	224.8	23 (14, 29)	1.01 (1, 1.02)	73 (64, 79)	32 (28, 34)	17 (4, 39)	5,671 (26.8)	772
Guangxi^b^ (ST)	1 (4)	6.6	22.9	108.4	2,349	96.2	22 (12, 28)	1 (0.99, 1.01)	79 (74, 83)	34 (32, 37)	12 (5, 27)	2,082 (31.5)	245
Guizhou^b^ (ST)	2 (5)	10.9	27.4	106.8	1,509	37.5	15 (4, 24)	0.9 (0.89, 0.9)	76 (73, 80)	33 (31, 36)	11 (4, 21)	1,920 (17.7)	179
Hainan^b^ (T)	5 (6)	4.1	19.6	110.1	2,818	93.5	25 (18, 29)	1 (1, 1.01)	79 (76, 82)	34 (32, 36)	16 (2, 36)	1,860 (45.9)	240
Hebei (WT)	4 (10)	33.7	38.1	115.8	3,598	21.7	14 (−2, 27)	1.01 (1, 1.02)	59 (45, 74)	26 (20, 33)	4 (0, 10)	1,899 (5.6)	344
Heilongjiang (MT)	3 (7)	18.6	46.1	126.2	3,286	22.4	5 (−15, 23)	0.99 (0.98, 1)	63 (50, 74)	27 (22, 33)	5 (1, 12)	1,634 (8.8)	198
Henan (WT)	2 (5)	13.2	34.7	113.1	3,015	98.2	15 (1, 26)	0.98 (0.97, 0.99)	61 (51, 79)	27 (22, 35)	5 (1, 11)	1,581 (11.9)	207
Hubei^b^ (ST)	4 (9)	21.1	30.9	112.6	3,320	41.7	17 (4, 28)	1 (0.99, 1.01)	74 (70, 78)	32 (30, 35)	11 (4, 19)	3,829 (18.2)	838
Hunan^b^ (ST)	3 (9)	14.2	27.4	113.0	2,990	92.5	18 (6, 30)	1 (0.99, 1.01)	73 (66, 78)	32 (30, 34)	15 (7, 25)	3,750 (26.5)	657
Mongolia (MT)	2 (6)	4.7	40.8	110.8	5,897	42.4	8 (−10, 24)	0.9 (0.89, 0.9)	49 (34, 59)	21 (15, 26)	3 (0, 7)	971 (20.5)	129
Jiangsu^b^ (ST)	4 (9)	24.2	32.9	118.6	6,550	75.7	16 (3, 29)	1.01 (1, 1.03)	73 (67, 80)	32 (29, 36)	10 (4, 19)	3,912 (16.2)	709
Jiangxi^b^ (ST)	3 (5)	11.4	28.2	115.3	2,538	58.0	19 (6, 30)	1.01 (1, 1.02)	75 (69, 78)	33 (30, 34)	17 (7, 30)	1,698 (15.0)	274
Jilin (MT)	3 (6)	14.0	44.1	125.4	3,893	40.2	6 (−15, 23)	0.99 (0.98, 1)	60 (46, 76)	26 (20, 34)	5 (1, 12)	1,805 (12.9)	321
Liaoning^b^ (WT)	7 (11)	26.5	40.7	122.6	5,158	34.0	10 (−7, 24)	1.01 (1, 1.02)	63 (52, 80)	27 (21, 36)	5 (1, 13)	3,409 (12.9)	341
Ningxia (MT)	4 (5)	4.8	37.6	106.0	3,188	24.7	10 (−7, 23)	0.87 (0.87, 0.88)	52 (37, 65)	23 (16, 28)	2 (0, 5)	1,122 (23.3)	164
Qinghai (C)	3 (5)	4.0	36.6	101.8	2,848	23.4	5 (−8, 17)	0.76 (0.75, 0.76)	56 (43, 71)	24 (18, 31)	4 (0, 9)	611 (15.2)	64
Shaanxi (WT)	2 (7)	12.8	34.3	108.8	3,175	59.2	10 (−4, 22)	0.88 (0.87, 0.89)	70 (55, 85)	30 (24, 37)	5 (1, 13)	1,258 (9.8)	309
Shandong (WT)	3 (7)	19.6	36.3	118.4	5,254	109.2	11 (−2, 23)	0.95 (0.94, 0.96)	64 (53, 82)	28 (23, 37)	8 (1, 22)	1,686 (8.6)	372
Shanghai^b^(ST)	1 (5)	18.2	31.3	121.5	11,563	4.7	18 (5, 29)	1.02 (1, 1.03)	72 (68, 76)	31 (29, 33)	12 (4, 20)	1,897 (10.5)	500
Shanxi (WT)	3 (5)	7.9	37.8	112.8	3,150	45.5	11 (−4, 25)	0.93 (0.92, 0.94)	57 (43, 73)	25 (19, 32)	4 (0, 8)	1,012 (12.7)	216
Sichuan^b^(ST)	4 (5)	18.3	30.2	104.0	2,538	111.6	18 (9, 26)	0.93 (0.92, 0.94)	70 (59, 78)	30 (25, 34)	11 (2, 24)	1,021 (5.6)	187
Tianjin^b^ (WT)	1 (5)	9.7	39.2	117.2	9,160	23.5	13 (−3, 27)	1.02 (1, 1.03)	60 (48, 74)	26 (21, 33)	4 (0, 11)	1,342 (13.8)	316
Xinjiang (MT)	2 (4)	4.0	43.8	87.6	2,919	81.6	7 (−15, 23)	0.92 (0.91, 0.93)	58 (43, 78)	25 (18, 34)	2 (1, 3)	1,271 (31.6)	202
Yunnan^b^ (ST)	4 (6)	19.1	24.8	103.0	1,982	17.8	17 (10, 21)	0.83 (0.82, 0.83)	68 (55, 78)	30 (23, 35)	9 (2, 22)	2,801 (14.7)	285
Zhejiang^b^ (ST)	4 (8)	22.2	30.0	120.4	6,535	97.7	18 (5, 30)	1.01 (1, 1.02)	72 (70, 75)	31 (30, 33)	14 (6, 19)	3,288 (14.8)	672

a Number of cities and hospitals participating in surveillance.

b Indicates year-round influenza surveillance before 2009 (all provinces switched to year-round surveillance in the post-2009 pandemic period).

C, cold temperate; GRP, gross regional product; MT, mid-temperate; ST, subtropical; T, tropical; WT, warm temperate;

We obtained daily meteorological data for each participating city during the study period, including temperature (minimum, maximum, mean), vapor pressure (minimum, maximum, mean), relative humidity (minimum, maximum, mean), rainfall, and hours of sunshine, as recorded by China Meteorological Administration (Table 1; Text S1) [22]. Province-level meteorological indicators were calculated as population-weighted averages of city-level data. Summary climate indicators were obtained by averaging the daily values of each climate factor by season (winter, Dec–Feb; spring, Mar–May; summer, Jun–Aug; fall, Sep–Nov), as well as calculating annual minimums and maximums. We also categorized the 30 provinces into six climatic zones on the basis of previous work [23], ranging from tropical to cold-temperate climates (Figure 1; Table 1).

### Estimates of Seasonal Characteristics

To visualize the average seasonal signature of influenza in each province, we first estimated the proportion of influenza cases identified in each week of the respiratory season, averaged across all complete years available for study. This method provided an empirical measure of seasonality, while adjusting for differences in sampling intensity and viral activity over time and between provinces [1],[24].

Weekly province-level influenza virus positive isolates were standardized by the annual number of influenza specimens tested prior to further modeling [25]. Preliminary analyses using a wavelet approach [26],[27] did not reveal changes in periodicity over time, so we elected to use stationary methods to characterize influenza seasonality. To obtain quantitative seasonality estimates, we fitted multiple linear regression models to weekly influenza time series separately in each province, including harmonic terms representing annual and semi-annual periodicities (see Text S1 for full details and [4],[26],[28],[29]). Briefly, the model follows:
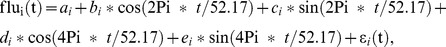
where flu_i_(t) are the weekly standardized counts of influenza positive A isolates (or B, or A+B combined) in province *i*; *t* is a running index for week; *a_i_*, *b_i_*, *c_i_*, *d_i_*, and *e_i_* are the intercept and seasonality coefficients to be estimated from the data; and ε*_i_*(t) are normally distributed errors.

On the basis of the estimated model coefficients representing harmonic terms, we extracted the amplitude of annual and semi-annual periodicities (*AnnAmp_i_* = sqrt(*b_i_* ^2^+*c_i_* ^2^) and *SemiAnnAmp_i_* = sqrt(*d_i_* ^2^+*e_i_* ^2^)), and the annual peak timing (*AnnPeakTiming_i_* = −atan(*c_i_*/*b_i_*)). To control for different levels of influenza activity across provinces, we calculated the *relative* amplitudes of annual and semi-annual periodicities, obtained by dividing *AnnAmp_i_* and *SemiAnnAmp_i_* estimates by the mean of the *flu_i_*(t) time series [26]. To estimate the relative contribution of semi-annual periodicity, we calculated the ratio between the amplitude of the semi-annual periodicity and the sum of the amplitudes of annual and semi-annual periodicities (*ratio_i_* = *SemiAnnAmp_i_/(AnnAmp_i_*+*SemiAnnAmp_i_*)). A ratio close to 1 is indicative of dominant semi-annual periodicity while a ratio close to 0 indicates dominant annual periodicity. Confidence intervals on estimates of relative amplitude, peak timing, and periodicity ratio were obtained by fitting seasonal regression models to 1,000 datasets resampled from the original data by block-bootstrap, which accounts for auto-correlation in weekly influenza incidences (Text S1).

As a sensitivity analysis, we fitted joint seasonal regression models in all 30 provinces using mixed effects models, accounting for fixed effects for broad geographic regions and random effects for provinces (Text S1). This approach revealed nearly identical seasonal curves as in the province-stratified analysis, indicating that the information contained in province-specific influenza data was sufficient to fit separate models. In the remainder of the paper, we report the results of province-stratified analyses. Further sensitivity analyses were conducted by fitting logistic regression models to the weekly influenza percent positive (weekly number of positive/weekly number specimens tested), which has also been used in past influenza research (Text S1) [27],[30].

In addition to seasonal parameters derived from regression models, we also quantified the median epidemic duration in each of the 30 provinces, defined as the number of weeks in which the reported number of influenza viruses exceeded a relative threshold, set at 2.5% or 5% of the total number of influenza viruses reported during the respiratory season. As a sensitivity analysis we estimated epidemic duration on the basis of the weekly percent positive exceeding an absolute threshold, set at 5% or 10% of all specimens tested in the week. We also assessed whether duration estimates were affected by sampling scheme, in particular whether surveillance was conducted year-round or limited to the October–March period.

As all analyses revealed important differences in the seasonality of influenza A and B, we present influenza A- and B-specific seasonal parameter estimates in the main text and refer to the Text S1 for aggregate analyses.

### Predictors of Influenza Seasonality

Next, we searched for predictors of influenza seasonal characteristics, including geography (latitude, longitude), population size and density, human mobility patterns, surveillance intensity (number of viruses sampled, number of participating hospitals and cities), and climate variables (Table 1). As seasonal characteristics were not fixed parameters but rather parameters estimated from seasonal regression models, we used a hierarchical Bayesian approach with non-informative priors to regress seasonal parameters against putative predictors (Text S1). A subset of predictors was first identified by classical stepwise multivariate analysis and these predictors were then used in the Bayesian approach. The errors obtained by bootstrap resampling were considered as observation variances in the Bayesian approach.

### Epidemiological Regions Relevant for Control

To assist with the design of routine influenza vaccination programs in China, in particular with regard to the optimal timing of vaccination, we set out to identify broad regions that share similar influenza epidemiological patterns. We applied hierarchical clustering using Ward's minimum variance method [31] to identify regional clusters, relying on the squared Euclidian pairwise difference between standardized influenza time series as the distance metrics [32]. We also performed sensitivity analyses using an alternative distance metric (Manhattan distance [32]) and clustering approach (complete linkage [33]). Finally, we applied stepwise linear discriminant analysis to identify the putative geographic, demographic, and climate predictors of the epidemiological regions defined by the cluster analysis.

## Results

### Sampling Intensity

During the study period 2005–2011, the average number of samples tested for influenza averaged 2,200 annually by province, with most intense sampling in Guangdong province in Southern China (5,661 samples per year) and thinnest sampling in Qinghai province in Northwest China (611 samples per year; Table 1). This level of sampling corresponds to 1.81 respiratory samples tested on average per year per 10,000 population in China (range across provinces 0.56–4.58). On average, 371 influenza virus positive specimens were identified annually by province (range across provinces 64–838).

### Influenza Seasonal Characteristics by Province

#### Empirical seasonality patterns and seasonal regression models

**Figure 2:**
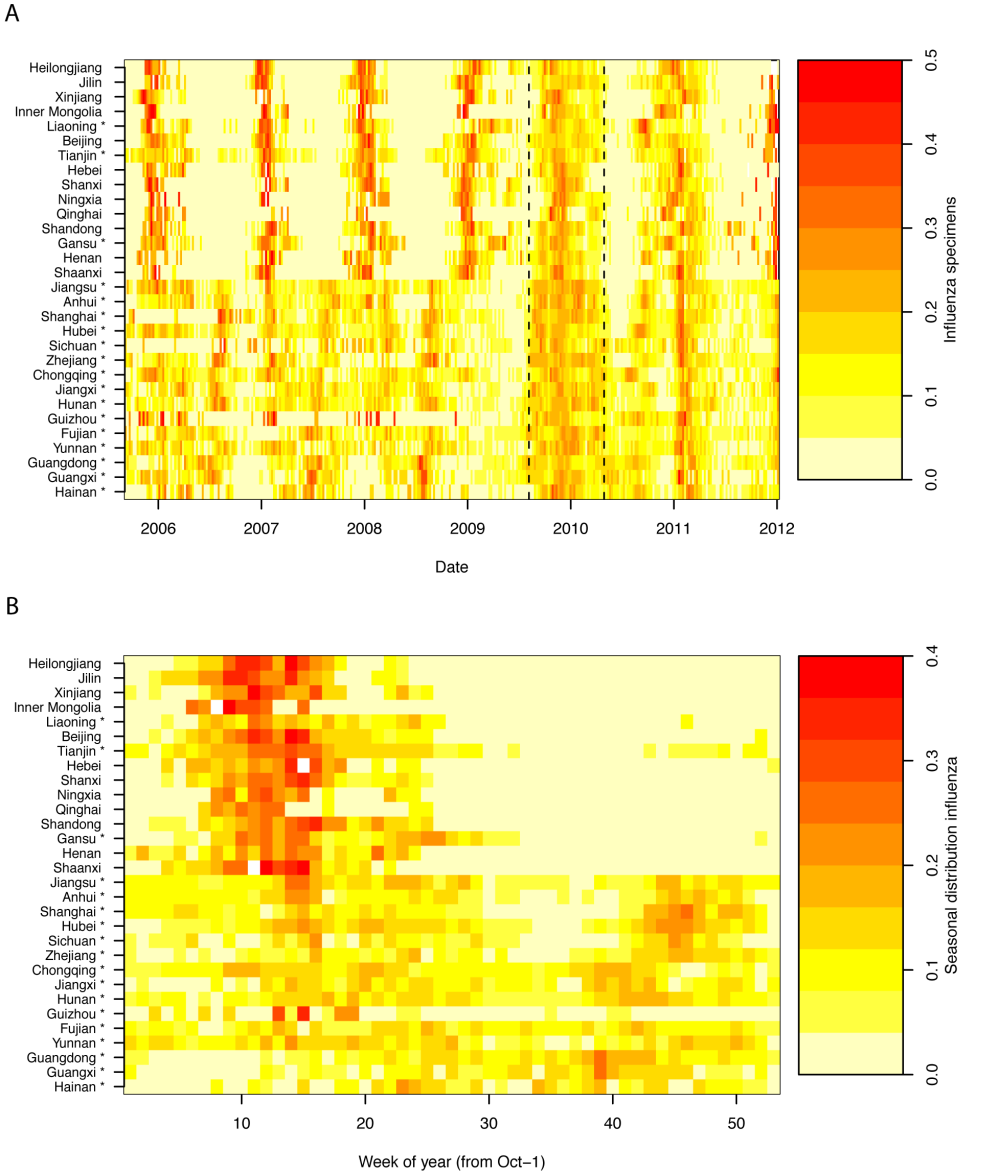
Heatmaps of influenza epidemiological data by Chinese province, Oct 2005-Dec 2011. (A) Time series of weekly standardized influenza cases, sorted by increasing latitude from bottom to top. Dashed vertical lines represent the influenza A/H1N1pdm pandemic period, Apr 2009–Apr 2010. (B) Average seasonal distribution of influenza cases (excluding the pandemic period), plotted as the proportion of viruses isolated in each week of the year. Provinces conducting year-round surveillance are denoted by an asterix. Week 0 is the first week of October of each year.

Heatmaps representing weekly province-level laboratory-confirmed influenza time series and their empirical seasonal signature are provided in Figure 2, revealing a diversity of seasonality patterns across China. While northern China experienced epidemics concentrated in winter, and southernmost provinces experienced influenza activity in spring–summer, provinces at intermediate latitudes did not exhibit clear annual seasonality.

Seasonal regression of time series data allowed further quantification of influenza seasonal characteristics and confirmed important differences by geography and virus type. Seasonal models fitted reasonably well for all influenza subtypes combined, influenza A, and high latitude provinces (median R^2^ = 23%, range 3%–60%); however fit was poorer for influenza B (median R^2^ = 10%, range 0.1%–26%; see Figures S1 and S2 and Text S1 for time series plots and residuals). Model fit however was not related to sampling intensity for any of the influenza outcomes (*p*>0.19; Text S1).

#### Periodicity

**Figure 3:**
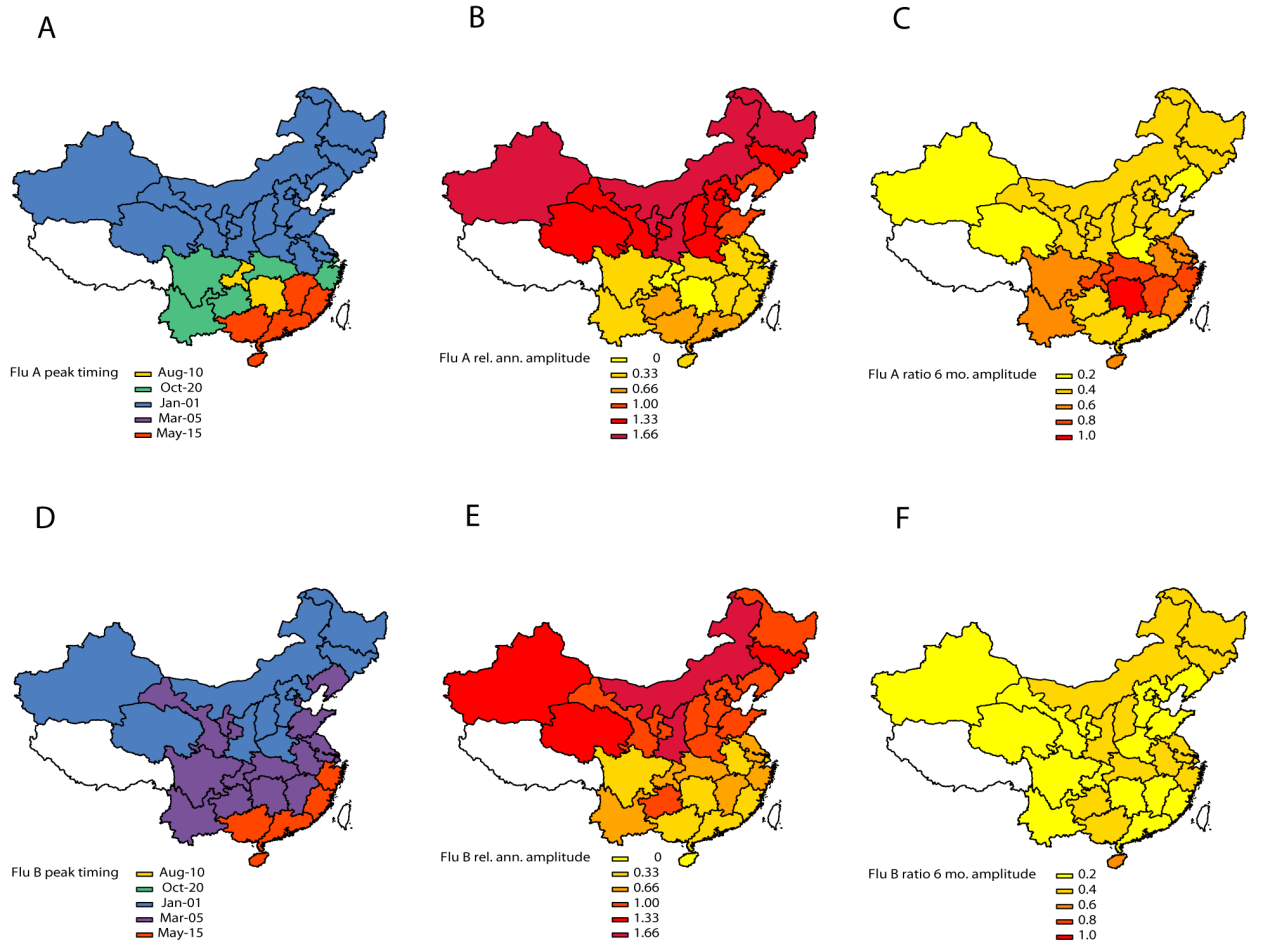
Estimates of periodicity and timing of influenza A (top panels) and B (bottom panels) epidemics in China. (Left) Timing of annual influenza peaks, in weeks. Timing is color coded by season. (Center) Amplitude of annual periodicity, ranging from low (yellow) to high (red), as indicated in the legend. Amplitude is relative to the mean of the weekly influenza time series in each province. (Right) Importance of semi-annual periodicities, measured by the ratio of the amplitude of the semi-annual periodicity to the sum of the amplitudes of annual and semi-annual periodicities. Yellow indicates strongly annual influenza epidemics, while red indicates marked semi-annual activity. See also Figure 4.

**Figure 4:**
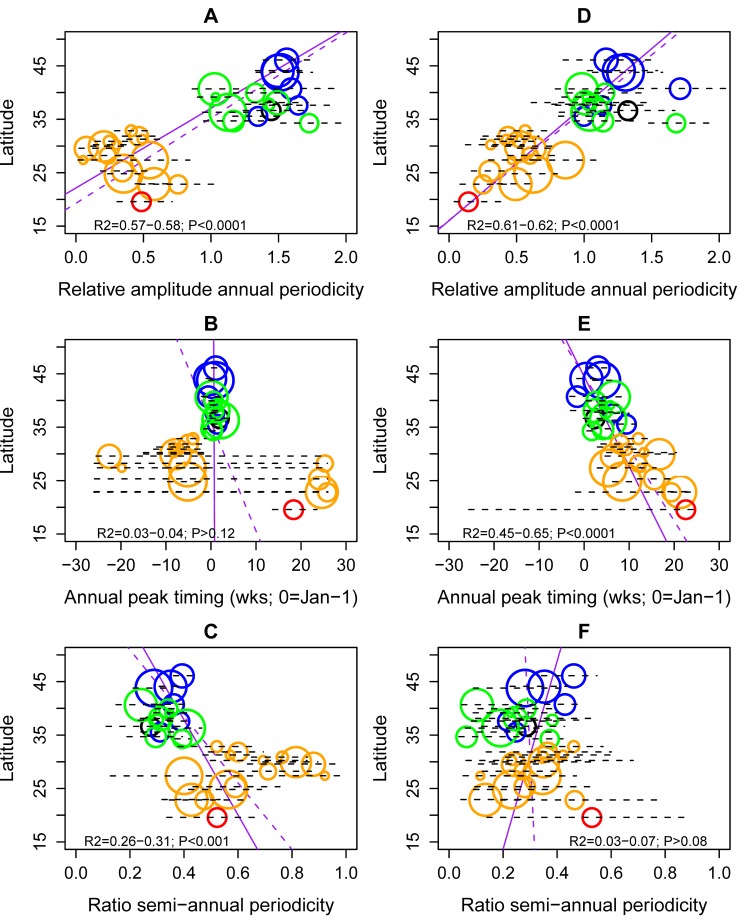
Latitudinal gradients in seasonality of influenza A and B epidemics in China. Plots represent estimates of seasonal parameters as a function of latitude for influenza A (left panels) and influenza B (right panels). Top panels: Amplitude of annual periodicity, relative to the mean standardized influenza time series. Middle panels: Peak timing in weeks. Bottom panels: Contribution of the semi-annual cycle, measured by the ratio of the amplitude of the semi-annual cycle to the sum of the amplitudes of annual and semi-annual cycles. Open circles represent point estimates from seasonal regression models and horizontal dashed lines represent 95% confidence intervals based on 1,000 block-bootstrap samples. Symbol size is proportional to the average number of influenza virus isolates sampled each season, while colors represent different climatic zones (black, cold-temperate; blue, mid-temperate; green, warm-temperate; orange, subtropical; red, tropical). Purple lines represent linear regression of seasonal parameters against latitude (dashed line, unweighted regression; solid line, regression weighted by the inverse of the variance of province-specific seasonal estimates); R^2^ and *p*-values are indicated on the graphs.

Influenza A displayed strong annual periodicity in provinces above ∼33°N latitude, and weaker annual periodicity at lower latitudes (median relative amplitude of annual cycle, 140% [95% CI 128%–151%] in the 15 northern temperate provinces versus 37% [95% CI 27%–47%] in the 15 southern provinces, Wilcoxon test, *p*<0.0001; Figure 3). Further, there was a strong latitudinal gradient in the amplitude of annual influenza A periodicity (R^2^ = 0.57; *p*<0.0001; Figure 4). Estimates of the periodicity of influenza B epidemics revealed a similar latitudinal gradient of higher annual amplitude in northern provinces (R^2^ = 0.61; *p*<0.0001; Figure 4). There was no difference in the amplitude of annual periodicity by influenza type (Wilcoxon paired test, *p* = 0.27).

The relative importance of influenza A semi-annual cycle also varied geographically, with highest contribution in seven mid-latitude provinces ranging from 27.4°N–31.3°N (ratio >0.6) (Figures 3 and 4). Overall, there was weak latitudinal gradient in importance of the semi-annual cycle, indicative of more intense semi-annual influenza A activity in southern China (slope −0.016 [95% CI −0.025 to −0.008], R^2^ = 0.31, *p*<0.001; Figure 4). In contrast, semi-annual periodicities were less pronounced for influenza B than for influenza A (*p*<0.0001 for paired difference in ratio of semi-annual cycle; Figure 4), with no latitudinal gradient in influenza B semi-annual periodicities (*p* = 0.77).

#### Peak timing

Influenza A peak timing estimates were concentrated in winter months in the 15 northern temperate provinces (median peak timing Jan 7; range Dec 24–Jan 15; Figures 3 and 4). In contrast, epidemic timing was more variable in the 15 tropical and subtropical southern provinces. Mid-latitude provinces experienced semi-annual peaks of influenza A in Jan–Feb and Jun–Aug, while the southernmost provinces had one major peak in spring (peak timing range, May 15 to Jun 15; Figure 3). There was no latitudinal gradient in influenza A epidemic timing (*p* = 0.12; Figure 4). In contrast, there was less geographic variation in seasonality of influenza B, with a distribution of estimated peaks of influenza B activity concentrated in colder months (Dec–Apr), except for three southern provinces experiencing peaks in May (Figures 3 and 4). Further, influenza B epidemics occurred gradually later in the year in provinces closer to the equator (R^2^ = 0.65, *p*<0.0001). Seasonal estimates for total influenza activity mirrored those found for influenza A, which accounted for 74% of all influenza viruses during the study period (Figures S3 and S4). Sensitivity analyses based on the weekly percent positive revealed similar seasonal patterns by geography and virus type (Figure S5).

#### Epidemic duration

We estimated relative and absolute measures of epidemic duration, which were highly correlated with each other for both influenza types (rho>0.79; *p*<0.0001). Average duration of influenza A epidemics was longer in southern than in northern provinces based on the relative epidemic threshold set at a fraction of the annual number of cases (medians 14.3 versus 10.8 wk for a 2.5% threshold, Wilcoxon *p*<0.0001; Figure S6). The pattern of longer epidemic duration in the south was robust to using an absolute threshold for the weekly proportion of respiratory specimens testing positive for influenza (medians 22.5 versus 15.6 wk for a 5% percent positive threshold, Wilcoxon *p* = 0.0002). Duration of influenza A epidemics increased with latitude based on both measures (R^2^ = 11%–37%, *p*<0.04; Figure S6). Similarly, influenza B epidemics were characterized by longer duration in southern provinces (13.2 versus 9.2 wk, *p*<0.0001; latitudinal gradient R^2^ = 11%–48%, *p*<0.04; Figure S6).

Estimates of epidemic duration were not affected by the surveillance scheme, with no difference between northern provinces conducting surveillance during Oct–Mar and those with year-round sampling (difference <1.0 wk, *p*>0.24). Differences in epidemic duration by geography and virus type were robust to using more conservative epidemic thresholds (i.e., using higher thresholds, resulting in shorter duration estimates).

#### Prevalence of influenza A and B

**Figure 5:**
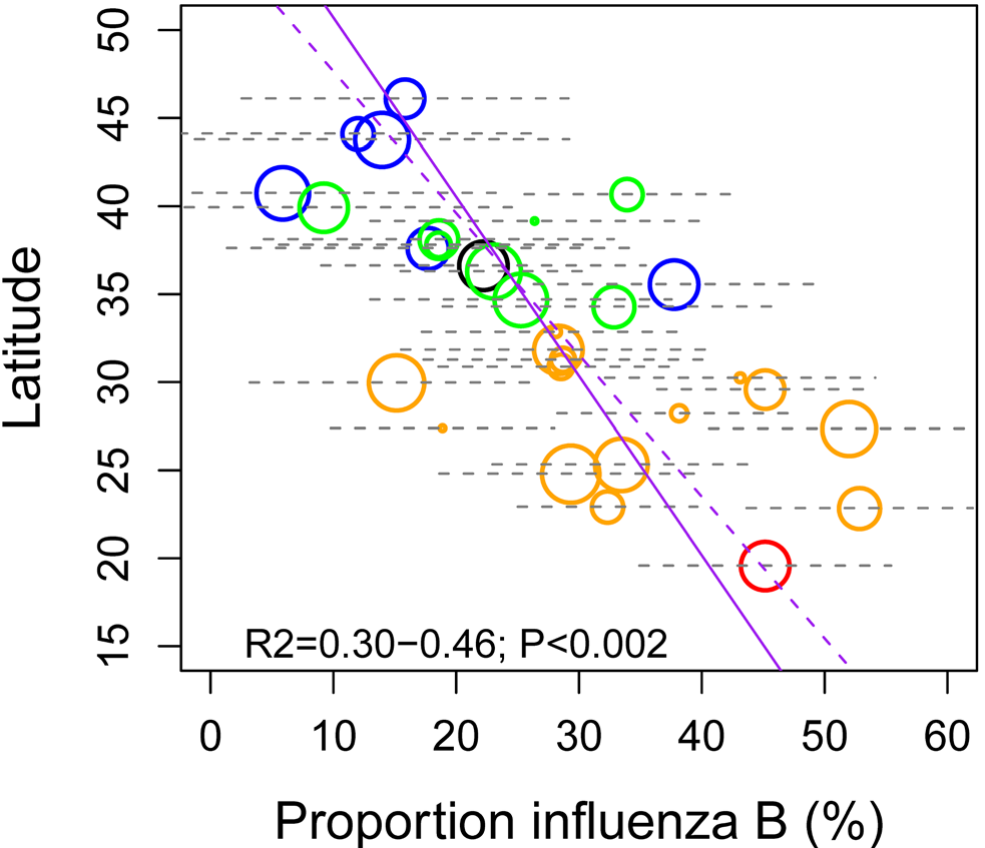
Latitudinal gradient in the dominance of influenza B in China, measured by the proportion of influenza B among all influenza positive isolates each season. Median proportion of influenza B over seven seasons is displayed for each province, with grey horizontal bars indicating ±2 standard deviations. Symbol size is proportional to the average number of influenza virus isolates sampled each season. Colors represent different climatic zones (black, cold-temperate; blue, mid-temperate; green, warm-temperate; orange, subtropical; red, tropical). Purple lines represent linear regression of influenza B proportion against latitude (dashed line, unweighted regression; solid line, regression weighted by sample size).

Given observed differences in the seasonality of influenza by geography and virus type, we checked whether the relative predominance of these viruses also differed across China. We found that the median proportion of influenza B among all influenza virus positive specimens ranged between 5%–55% across provinces in the 7 study years, with increasing prevalence towards the south (Spearman rho = −0.71 between latitude and influenza B proportion; *p*<0.0001; Figure 5).

### Predictors of Influenza Seasonality

Next, we searched for climate, demographic, or geographic predictors of influenza seasonal characteristics using a hierarchical Bayesian approach, while adjusting for differences in sampling intensity between sites (Table 2). Climate variables and latitude were typically the most strongly associated with influenza seasonal characteristics. In particular, stronger annual amplitude of epidemics was associated with lower minimum temperature, for all viruses combined and analyses stratified by influenza type (range across models for different influenza outcomes, R^2^ = 64%–76%, *p*<0.001). Stronger semi-annual cycles and longer epidemic durations were associated with lower minimum amounts of sunshine (R^2^ = 32%–51%, *p* = 0.01), for all influenza and influenza A. Predictors of influenza peak timing were more diverse, with maximum amount of rainfall explaining later influenza activity in spring months for all viruses combined. In contrast, earlier timing of influenza B was strongly associated with higher latitude (R^2^ = 72%, *p*<0.0001); while there was no relationship for influenza A. Mobility patterns were weakly but significantly associated with total influenza seasonal characteristics, explaining 5%–16% of the variance in peak timing and annual periodicity, while population density explained 7%–12% of peak timing and duration. No predictor was identified for influenza A peak timing or for influenza B duration and periodicity ratio. Variables related to surveillance design and sampling intensity explained less than 13% of the variance in seasonal characteristics in best-fit models, a typically lower proportion than climatic variables.

**Table 2:** Predictors of influenza seasonal characteristics by virus type (influenza A, B, and total) in 30 Chinese provinces, 2005–2011.

Annual Periodicity			Annual Peak Timing			Periodicity Ratio^a^			Epidemic Duration^b^		
Predictor	Estimate (SE)	Partial R^2^	Predictor	Estimate (SE)	Partial R^2^	Predictor	Estimate (SE)	Partial R^2^	Predictor	Estimate (SE)	Partial R^2^
***Influenza A***											
Minimum temperature (°C)	−0.0029 (0.0009)	0.64	None^c^			Minimum hours of sunshine (h)	−0.008 (0.0016)	0.51	Minimum hours of sunshine (h)	−0.147 (0.038)	0.29
Minimum hours of sunshine (h)	0.0202 (0.0051)	0.11				Total *n* influenza isolates	3.1E–05 (1.4E–05)	0.09	Per capita GRP	5.2E–04 (2.5E–04)	0.11
*n* participating cities	−0.082(0.042)	0.04									
***Influenza B***											
Minimum temperature (°C)	−0.0048 (0.0005)	0.76	Latitude	−0.412 (0.097)	0.72	None^c^			None^c^		
			Total *n* specimens tested	3.2E–04 (6.4E–05)	0.08						
***Influenza A and B combined***											
Minimum temperature (°C)	−0.005 (0.0008)	0.68	Maximum rainfall (mm)	0.30 (0.07)	0.42	Minimum hours of sunshine	−0.0048 (0.0019)	0.32	Minimum hours of sunshine (h)	−0.216 (0.061)	0.37
Total *n* influenza isolates	−1.4E–04 (4.5E–05)	0.05	Boat travel^d^	−0.002 (0.0015)	0.16	Total *n* influenza isolates	4E–05 (1.8E–05)	0.13	Population density (pop/km^2^)	0.0050 (0.0019)	0.12
Air travel^d^	−1.2E–04 (6E–05)	0.05	Population density (pop/km^2^)	−0.0041 (0.0019)	0.07						

Putative predictors include climate, geographic, population, and sampling factors. Seasonal characteristics were based on linear seasonal regression models fitted to province-specific weekly laboratory surveillance data (Figure 4). Point estimates and standard errors provided in the table are based on hierarchical Bayesian regression of seasonal characteristics against province-specific covariates, with observation variances estimated by bootstrap resampling of seasonal parameters.

a Amplitude of semi-annual cycle divided by the sum of amplitudes of semi-annual and annual cycles.

b 5% threshold criterion.

c No covariate remained in final model.

d Annual number of passengers leaving or entering the province, divided by province-specific population size.

### Defining Broad Influenza Epidemiological Regions Relevant for Vaccination

**Figure 6:**
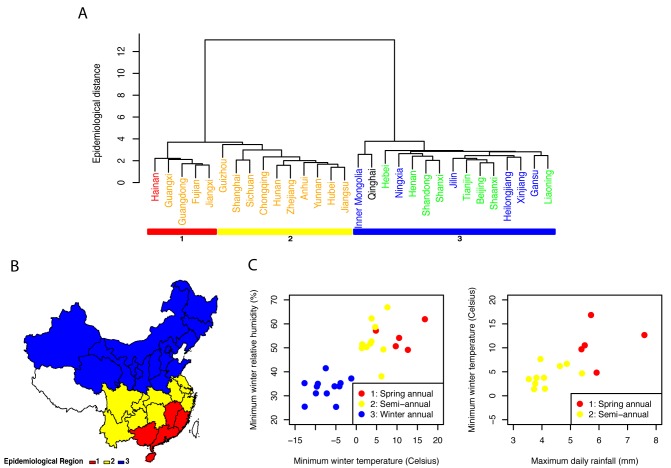
Influenza epidemiological regions and climate predictors. (A) Definition of three influenza epidemiological regions based on hierarchical clustering (colored rectangles; Ward's method, Euclidian distance between weekly standardized influenza time series). Province labels are color-coded by climatic region (black, cold-temperate; blue, mid-temperate; green, warm temperate; orange, subtropical; red, tropical). (B) Map of the three epidemiological regions identified in (A). (C) Climate predictors of the two main regions identified in (A) (subtropical regions 1 and 2 versus temperate region 3), based on stepwise discriminant analysis. (D) Climate predictors of subtropical regions 1 and 2, based on stepwise discriminant analysis.

Next, we identified broad regions that share similar influenza epidemiology and could be used in the design of geographically-tailored immunization campaigns. Although our results suggest differences in the seasonality of influenza by virus type, vaccine policies are typically set on the basis of the sum of influenza A and B activity. Hence we applied hierarchical clustering algorithms to province-level time series for influenza A and B combined in the main analysis (Figure 6) and present type-specific regions in Figure S7 and Text S1. The analysis of total influenza activity revealed two main regions that aligned with broad climatic zones (northern temperate versus southern [sub]tropical provinces; Figure 6A and 6B). Within the cluster of southern provinces (*n* = 15), we identified two subclusters coinciding with the mid-latitude region that experienced semi-annual influenza activity (*n* = 10), and the southernmost region where influenza activity was concentrated in spring (*n* = 5).

The same approach stratified by virus type revealed similar regions for influenza A than for total influenza; while influenza B regions were more different and less climatologically structured (Figure S7). We also considered alternative clustering and distance methods (Figure S8): overall the separation between mid-latitude provinces and southernmost provinces was consistent across algorithms. Two mid-latitude provinces however, Chongqing and Guizhou, did not consistently cluster in the same epidemiological group, suggesting that more information will be needed from these provinces.

Stepwise discriminant analysis of the two main influenza epidemiological clusters revealed that winter minimum temperature and relative humidity were the two strongest predictors of whether influenza exhibited winter seasonality (north) or more complex seasonal patterns (south) (Figure 6C). The predictive accuracy of discriminant models including these two climatic variables was 100% by leave-one-out cross-validation. Discriminant analysis restricted to the two southern subclusters indicated that maximum rainfall and minimum winter temperature were predictive of influenza seasonality differences in this region. Rainier and warmer provinces experienced a dominant annual influenza peak in spring (Figure 6D) with 93% predictive accuracy of discriminant models by leave-one-out cross-validation. None of the other climate, population, or mobility factors were significantly associated with influenza epidemiological clusters.

## Discussion

To our knowledge, this is the first comprehensive study of influenza seasonal characteristics by virus type and geography in China. On the basis of multiyear laboratory-confirmed influenza surveillance data representative of a large majority of the Chinese population, we identified three epidemiological regions characterized by distinct seasonality: northern provinces (latitudes >33°N) experience winter epidemics, southernmost provinces (latitude <27°N) experience peak activity in spring, while provinces at intermediate latitudes experience semi-annual epidemic cycles. Two complementary statistical approaches indicated that cold temperatures were predictive of the occurrence of influenza in winter in the north and of the strength of the annual periodicity, with some contributing effect of relative humidity, while precipitations were linked to influenza activity in spring in Southern China. These marked geographic differences in influenza seasonality are important to consider for routine influenza immunization campaigns in China, as they entail different timings of vaccination, and use of potentially different vaccine compositions.

China is a large country encompassing a diversity of climatic zones and hence it is perhaps not surprising that our results support a diversity of influenza seasonal patterns. The latitudinal gradients in timing, amplitude, and duration of influenza activity evidenced in this study are reminiscent of those reported in another large country, Brazil, where epidemics originate in the northern equatorial states and move towards the subtropical south over a 3-month period [26]. Similar gradients have been observed on a global scale [3],[34].

The epidemiology of influenza in China and Brazil [26] contrasts with the hierarchical spread of the disease in the US, where winter epidemics are driven by large population centers and typically highly synchronized, including in a subtropical city like Miami (latitude 25°N) [27]. It is likely that influenza seasonality results from the combined effect of climate factors and population mobility patterns [8],[26],[35], with potentially higher population mixing within the US than within Brazil or China. We did not find a strong association between seasonality and mobility patterns in our Chinese study, although our travel data did not include international statistics. Further evidence for the role of population mobility comes from the disconnect between influenza seasonality in neighboring regions sharing similar climate [35], including Hong-Kong, Special Administrative Region, which experiences semi-annual cycles [36], and Guangdong, China, where influenza activity peaks in the spring (this study and [37]). A recent phylogeographic comparison of influenza viruses circulating in Southern China did not reveal extensive viral migrations between these two locales [37], suggesting that mixing is perhaps not sufficient to synchronize epidemics. Similarly, analysis of historical influenza time series from Iceland highlights the importance of international connectivity in driving the timing and periodicity of epidemics [38].

As with previous large-scale analyses linking influenza and climatic drivers [2],[8], no single climatic variable captured the complexity of influenza seasonality patterns across China. However, minimum temperature, humidity, and precipitations helped distinguish between annual activity in winter or spring, and more complex seasonal patterns. The latitudinal threshold associated with influenza winter seasonality in our data, estimated at ∼33°N, is equivalent to minimum winter temperature threshold of ∼0°C. A recent climate-based model for influenza seasonality, derived from a much larger global sample of epidemiologic data, has proposed a different threshold at 18°C–21°C [8]. Further research should focus on whether local or regional factors beyond climate may affect influenza seasonality and should be incorporated in global models such as [8].

Our most intriguing finding was perhaps the marked differences in seasonality of influenza A and B viruses, with most of China experiencing annual influenza B activity in winter. This is the first report of such distinct epidemiological patterns in influenza A and B activity, although it has been noted previously that influenza B epidemics peak a few weeks later than influenza A epidemics on average in temperate regions [34]. Further, local studies have hinted at differences in the association between influenza and climate by virus type, including in Dakar, Senegal [39]. Experimental studies indicate that both influenza A and B transmit via aerosols and droplets, with colder temperature increasing duration of viral shedding and transmission of both viruses [40],[41]. The intriguing seasonality differences observed at the population level in our study could be driven by differences in mean age at infection, immunity, or persistence patterns between influenza A and B viruses [42]–[45].

The motivation for our study was to assist on-going efforts to strengthen influenza surveillance and research in China and develop a national immunization program. Substantial antibody titers decay within 4–8 months post-vaccination [46],[47], combined with rapid antigenic drift in circulating viruses [48], require vaccination campaigns to be initiated a few weeks prior to the onset of local influenza activity. A practical outcome of our study was to define three broad influenza regions on the basis of epidemiological data (Figure 6B), which have distinct influenza seasonality characteristics and broadly align with climatic zones. Optimization of vaccine policy in China is complicated by differences in seasonality of influenza A and B, especially in the southern region, so that it may not be possible for a single annual influenza vaccination campaign to offer optimal protection for both virus types. Influenza A represented 74% of all influenza viruses in our dataset, and hence it is not surprising that seasonality patterns aligned broadly for total influenza and influenza A activity. More refined type-specific vaccine policies could be defined on the basis of province-specific information on the relative dominance and disease burden of each virus. Interestingly, recent work indicates a higher excess mortality burden of influenza B epidemics in China, relative to that in other countries [11],[15]; however the underlying biological mechanism remains unclear and province-specific burden estimates are lacking.

In this context, our data indicate that it is optimal for Northern China to follow the timing of vaccination typically recommended for the Northern Hemisphere, with annual campaigns starting in October. In contrast, southernmost Chinese provinces have to accommodate influenza activity peaking in April–June, and hence vaccination would be best initiated in February–March of each year, broadly coinciding with the recommended timing of vaccination for the Southern Hemisphere. The relevance of using vaccine recommendations for the opposite hemisphere in some regions has previously been demonstrated in the case of Brazil [9].

The situation of mid-latitude Chinese provinces is more complex due to semi-annual patterns of influenza A activity and longer epidemic periods. While the results of hierarchical clustering and sensitivity analyses suggest that mid-latitude provinces should be considered separately from southernmost China (*p*<10^−9^ for seasonality differences between Guangdong and Guangxi provinces and other southern provinces in mixed models; Text S1), denser sampling over a longer time period could help further clarify the most sensible geographic breakdown. Of note, we were unable to robustly classify seasonal patterns in two mid-latitude provinces, Chongqing and Guizhou, perhaps due to low sample size, spatial aggregation, or border effects. More epidemiological data will be needed before a specific timing of vaccination can be recommended for these provinces. Other locales in the broader region have adopted the Northern Hemisphere vaccine [10], including Singapore, Vietnam, and Hong-Kong SAR, and a quantitative evaluation of vaccination strategies could be beneficial in areas experiencing semi-annual or year-round influenza circulation. Increased regional capacity for vaccine production in east Southeast Asia [10] provides a unique opportunity to design local vaccination strategies, rather than relying on traditional geographic divisions between Northern and Southern Hemisphere countries.

In China, seasonal influenza vaccination was introduced in 1998, with four international and ten domestic manufacturers now supplying trivalent inactivated vaccine to the Chinese market. Vaccine coverage has increased over time but it is still relative low at around 2% nationally [16]. With rapid economic development, we anticipate a rapid increase of influenza vaccine use in the private sector in the near future in China. Several wealthy cities have already introduced local government subsidy programs. In particular, Beijing has provided free annual influenza vaccination since 2007 to adults >60 y and school-age children, while Xi'an City in Shaanxi province and Ningbo City in Zhejiang province have provided annual influenza vaccines through government health insurance since 2004 and 2010, respectively. Vaccine coverage is expected to rise substantially with increasing data on national- and province-specific influenza disease burden [11],[15], seasonality, cost-effectiveness, and initiation of national and provincial-level immunization programs.

Our study is prone to a number of limitations. Our results are based on a relatively short period of time, 2005–2011, which limited our ability to capture multiyear influenza periodicities. Sampling intensity was not constant throughout time and could have affected our results, although data standardization and sensitivity analyses suggest this was not a major issue. Since a fraction of the northern provinces did not conduct year-round surveillance before 2009, it would be useful to revisit seasonality patterns in key provinces bordering the 33°N latitude threshold with longer year-round surveillance time series.

Our dataset was too coarse to evaluate influenza patterns at the city or hospital level. However, surveillance data were based on a single city for five of the 30 studied provinces (including two northern and three southern provinces; Table 1), and hence influenza surveillance in these five provinces is not subject to aggregation issues. In particular, two of these “single-city” provinces, Beijing and Tianjin, are immediate neighbors with nearly identical influenza patterns, suggesting that surveillance information from this dataset is robust and captures true geographical differences in disease dynamics. Since most of the influenza information came from relatively large cities, however, we were unable to assess the finer details of influenza spatial transmission, including potential differences between rural and urban areas. Previous work suggests that influenza activity in rural areas of Western China is generally synchronous with that of the more populous Eastern coastal areas [18].

We chose to exclude information pertaining the 2009 pandemic period to focus on the seasonality of inter-pandemic influenza, and it is unclear how the emergence of the pandemic virus perturbed seasonality of the resident (sub)types. However, sensitivity analyses limited to data from the pre-pandemic period confirmed our findings (not shown). Another caveat is the lack of information on influenza A/H1N1 seasonality, as sampling was too thin to explore A/H3N2 and A/H1N1 viruses separately, and the patterns reported here for influenza A reflect those of the dominant A/H3N2 subtype.

We have provided here a statistical description of influenza seasonality in China to inform timing of vaccination, using a two-stage approach used in past research to characterize the circulation of influenza and other infections in large regions and assess potential links with climate [2],[26],[49]–[51]. Our approach improves on previous work by integrating uncertainty in seasonal estimates obtained in the first stage analysis in hierarchical Bayesian models. However, our relatively simple seasonal models (see also [4]) explain only a fraction of the variance in weekly influenza surveillance data (typically ≤50% for influenza A and ≤30% for influenza B). Year-to-year variability in influenza epidemiology, complex virus circulation patterns specific to China, and sampling issues, may all contribute to weak model fit. Further, there was residual auto-correlation in some of our province-specific models, although auto-correlation was taken into account in error estimates via a block-bootstrap approach.

Further work could focus on fitting compartmental transmission model to evaluate the transmissibility of the virus in different regions of China, and assess differences in herd immunity thresholds for vaccination. Interestingly, the effective reproduction number of influenza is thought to be relatively similar between temperate and tropical countries, ranging from 1.1–1.4 [52], suggesting that background immunity and transmission dynamics are broadly similar across regions (although it likely varies between years [53]). Overall, more modeling work is needed to evaluate regional differences in the evolutionary and transmission dynamics of influenza and their association with climate and demographic factors [3],[8],[18],[54]–[56].

### Conclusion

In conclusion, our study uncovered intriguing differences in influenza seasonality between regions and virus types in China, some of which can be associated with climatic factors, and confirm previous reports from other regions [3],[26]. Further work should focus on quantifying the balance between climatic drivers, population mixing, and other factors affecting influenza seasonality patterns globally, which could differ by virus types and subtypes. Our work has practical implications for the design of routine immunization programs in China, and suggests the need for staggered timing of vaccination in three broad epidemiological regions. Further surveillance studies are warranted to confirm these seasonality patterns and assess the match between influenza strains circulating in different provinces and WHO vaccine recommendations [18]. As routine immunization campaigns are rolled out and local vaccine production improves in resource-limited regions, it will become increasingly important to ensure that vaccination strategies are optimally tailored to the local epidemiology of the disease.

## Supporting Information

Figure S1 **Fit of type-specific seasonal influenza models in three provinces representative of broad influenza epidemiological regions in China.** Shanxi (latitude 37.8°N, northern temperate province experiencing winter seasonal influenza A and B epidemics), Hubei (latitude 30.9°N, mid-latitude subtropical province experiencing semi-annual influenza A epidemics), and Guangdong (22.9°N, southern subtropical province experiencing late spring influenza A epidemics). Blue curve, observed cases standardized by the annual number of specimens tested; red curve, seasonal model. Grey lines mark Jan 1st of each year, while the green lines mark the 2009 A/H1N1 pandemic season, which was not included in the model fitting procedure. Model is based on a linear regression with harmonic terms for annual and semi-annual periodicities.
(TIFF)

Figure S2 **Residuals of seasonal models presented in Figure S1 in three selected provinces.**
(TIF)

Figure S3 **Estimates of periodicity and timing of influenza epidemics in China (A and B combined).** (Left) Timing of annual influenza peaks, in weeks. Timing is color coded by season. (Center) Amplitude of annual periodicity, ranging from low (yellow) to high (red), as indicated in the legend. Amplitude is relative to the mean of the weekly influenza time series in each province. (Right) Importance of semi-annual periodicities, measured by the ratio of the amplitude of the semi-annual periodicity to the sum of the amplitudes of annual and semi-annual periodicities. Yellow indicates strongly annual influenza epidemics, while red indicates marked semi-annual activity. See also Figure S4.
(TIF)

Figure S4 **Latitudinal gradients in seasonality of total influenza activity (A and B combined) in China.** Left: Relative amplitude of annual periodicity. Middle: Peak timing. Right: Contribution of the semi-annual cycle, measured by the ratio of the amplitude of the semi-annual cycle to the sum of the amplitudes of annual and semi-annual cycles. Open circles represent point estimates from seasonal regression models and horizontal dashed lines represent 95% confidence intervals based on 1,000 block-bootstrap samples. Purple lines represent linear regression of seasonal parameters against latitude (dashed line, unweighted regression; solid line, regression weighted by the inverse of the variance of province-specific seasonal estimates); R^2^ and *p*-values are indicated on the graphs. Colors represent different climatic zones (black, cold-temperate; blue, mid-temperate; green, warm-temperate; orange, subtropical; red, tropical).
(TIFF)

Figure S5 **Sensitivity analysis on seasonal estimates for influenza A (top) and B (bottom) using a different model structure.** Same as Figure 4 but using a logistic seasonal model with binomial errors to model weekly percent positive for influenza A and B (weekly number of influenza positive/weekly number of specimens tested). Left: Relative amplitude of annual periodicity. Middle: Peak timing. Right: Contribution of the semi-annual cycle, measured by the ratio of the amplitude of the semi-annual cycle to the sum of the amplitudes of annual and semi-annual cycles. Open circles represent point estimates from seasonal regression models. Purple lines represent linear regression of seasonal parameters against latitude; *p*-values are indicated on the graphs. Colors represent different climatic zones (black, cold-temperate; blue, mid-temperate; green, warm-temperate; orange, subtropical; red, tropical).
(TIF)

Figure S6 **Latitudinal gradient in duration of influenza epidemics, by province and virus type.** Duration is based on a relative measure (number of weeks with more than 2.5% of annual influenza virus isolated), or an absolute measure (number of weeks with more than 5% influenza percent positive). Top panels: influenza A and B combined; middle panels: influenza A; bottom panels: influenza B. Horizontal grey bars represent ±2 standard deviations based on inter-annual variability in the 7 study years.
(TIF)

Figure S7 **Influenza A (top) and B (bottom) epidemiological regions identified by cluster analysis.** Epidemiological regions are based on hierarchical clustering (Ward's method), using the Euclidian distance between weekly standardized influenza time series. Provinces are color-coded by climatic region (black, cold-temperate; blue, mid-temperate; green, warm-temperate; orange, subtropical; red, tropical).
(TIFF)

Figure S8 **Sensitivity analyses on cluster algorithms used to define influenza epidemiological regions (compare with** **Figure 6** **).** Top: using a different distance metric for pairwise differences between influenza time series (absolute distance, also known as Manhattan distance, instead of Euclidian distance). Bottom: using a different clustering algorithm (complete linkage instead of Ward). Analyses are based on total influenza activity.
(TIFF)

Text S1 **Description of supplementary information.**
(DOC)
