# Supplementary figures and images for: Characterization of Regional Influenza Seasonality Patterns in China and Implications for Vaccination Strategies: Spatio-Temporal Modeling of Surveillance Data
Source: PLoS Med. 2013 Nov 19;10(11):e1001552. doi: 10.1371/journal.pmed.1001552 (PMC3864611; doi:10.1371/journal.pmed.1001552)

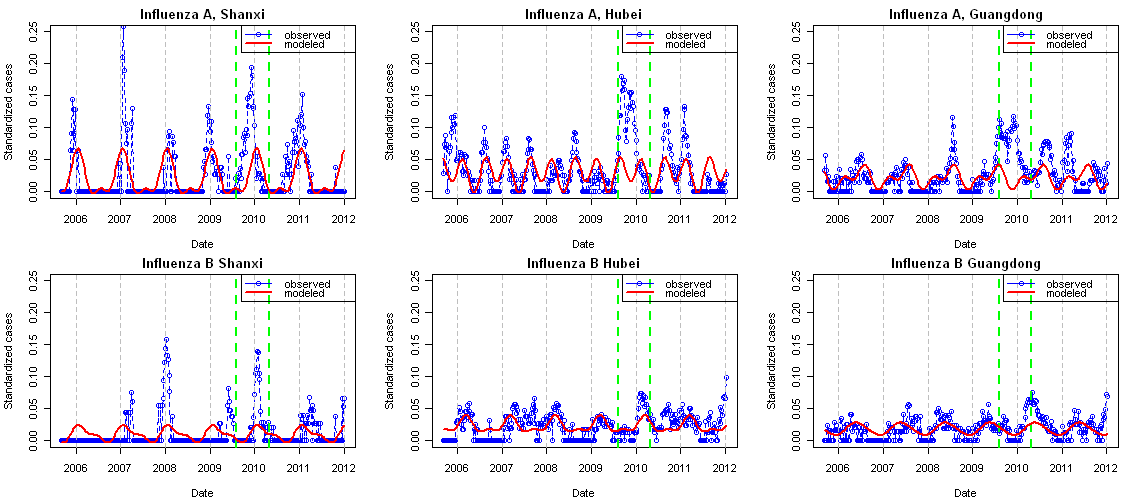

Supplement: Figure S1 — Fit of type-specific seasonal influenza models in three provinces representative of broad influenza epidemiological regions in China. Shanxi (latitude 37.8°N, northern temperate province experiencing winter seasonal influenza A and B epidemics), Hubei (latitude 30.9°N, mid-latitude subtropical province experiencing semi-annual influenza A epidemics), and Guangdong (22.9°N, southern subtropical province experiencing late spring influenza A epidemics). Blue curve, observed cases standardized by the annual number of specimens tested; red curve, seasonal model. Grey lines mark Jan 1st of each year, while the green lines mark the 2009 A/H1N1 pandemic season, which was not included in the model fitting procedure. Model is based on a linear regression with harmonic terms for annual and semi-annual periodicities. (TIFF) [file pmed.1001552.s001.tiff]

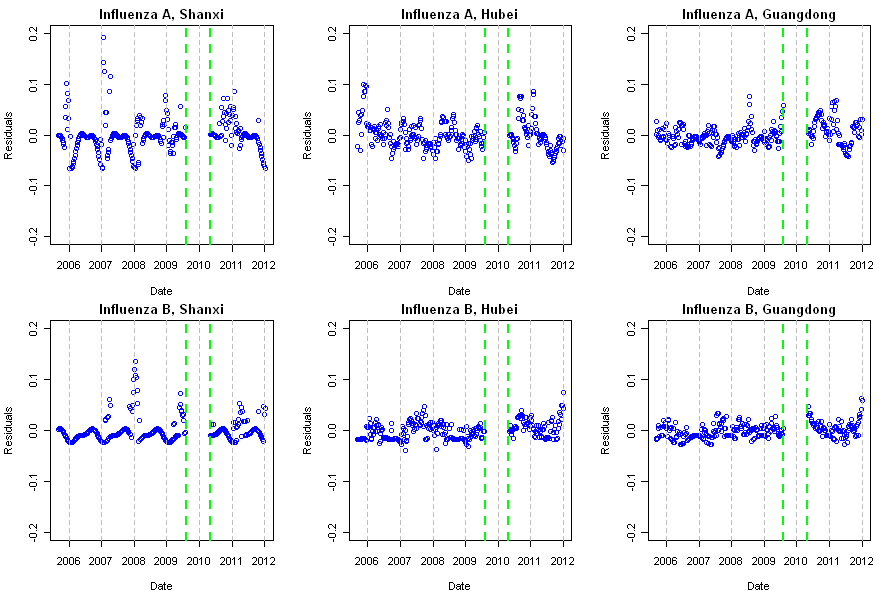

Supplement: Figure S2 — Residuals of seasonal models presented in Figure S1 in three selected provinces. (TIF) [file pmed.1001552.s002.tiff]

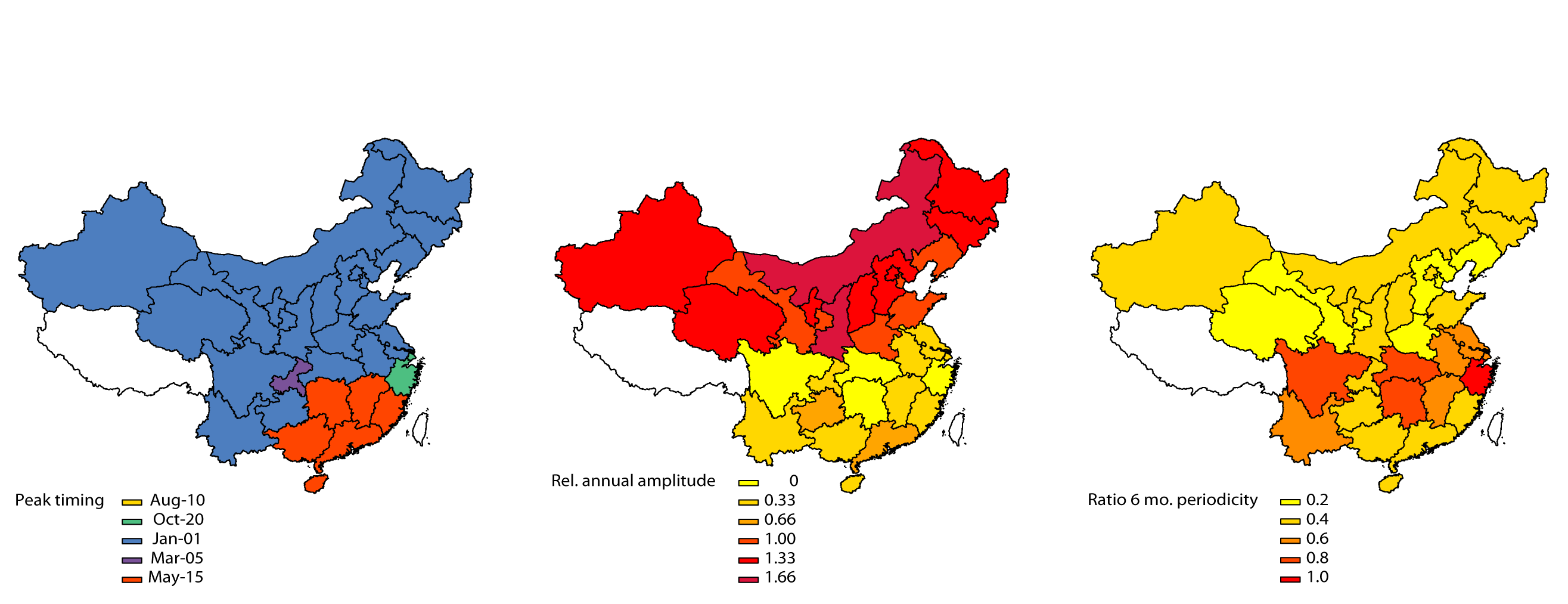

Supplement: Figure S3 — Estimates of periodicity and timing of influenza epidemics in China (A and B combined). (Left) Timing of annual influenza peaks, in weeks. Timing is color coded by season. (Center) Amplitude of annual periodicity, ranging from low (yellow) to high (red), as indicated in the legend. Amplitude is relative to the mean of the weekly influenza time series in each province. (Right) Importance of semi-annual periodicities, measured by the ratio of the amplitude of the semi-annual periodicity to the sum of the amplitudes of annual and semi-annual periodicities. Yellow indicates strongly annual influenza epidemics, while red indicates marked semi-annual activity. See also Figure S4. (TIF) [file pmed.1001552.s003.tiff]

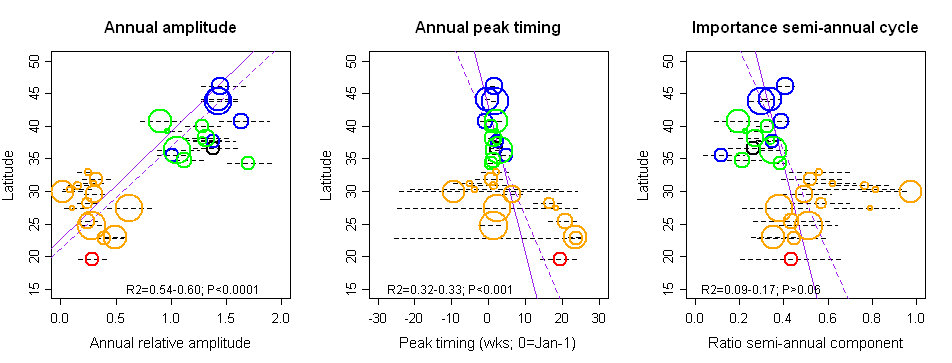

Supplement: Figure S4 — Latitudinal gradients in seasonality of total influenza activity (A and B combined) in China. Left: Relative amplitude of annual periodicity. Middle: Peak timing. Right: Contribution of the semi-annual cycle, measured by the ratio of the amplitude of the semi-annual cycle to the sum of the amplitudes of annual and semi-annual cycles. Open circles represent point estimates from seasonal regression models and horizontal dashed lines represent 95% confidence intervals based on 1,000 block-bootstrap samples. Purple lines represent linear regression of seasonal parameters against latitude (dashed line, unweighted regression; solid line, regression weighted by the inverse of the variance of province-specific seasonal estimates); R2 and p-values are indicated on the graphs. Colors represent different climatic zones (black, cold-temperate; blue, mid-temperate; green, warm-temperate; orange, subtropical; red, tropical). (TIFF) [file pmed.1001552.s004.tiff]

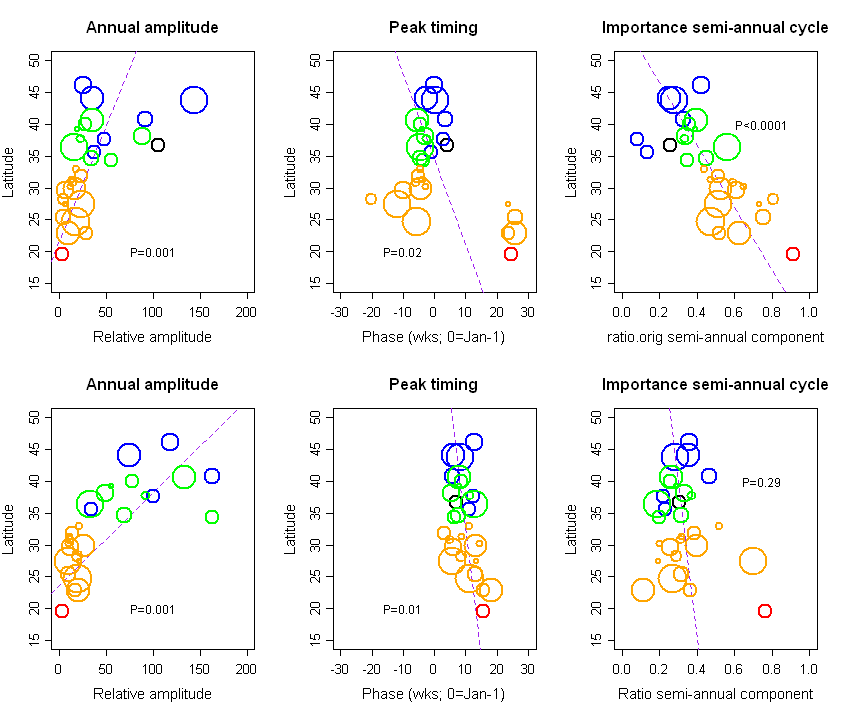

Supplement: Figure S5 — Sensitivity analysis on seasonal estimates for influenza A (top) and B (bottom) using a different model structure. Same as Figure 4 but using a logistic seasonal model with binomial errors to model weekly percent positive for influenza A and B (weekly number of influenza positive/weekly number of specimens tested). Left: Relative amplitude of annual periodicity. Middle: Peak timing. Right: Contribution of the semi-annual cycle, measured by the ratio of the amplitude of the semi-annual cycle to the sum of the amplitudes of annual and semi-annual cycles. Open circles represent point estimates from seasonal regression models. Purple lines represent linear regression of seasonal parameters against latitude; p-values are indicated on the graphs. Colors represent different climatic zones (black, cold-temperate; blue, mid-temperate; green, warm-temperate; orange, subtropical; red, tropical). (TIF) [file pmed.1001552.s005.tif]

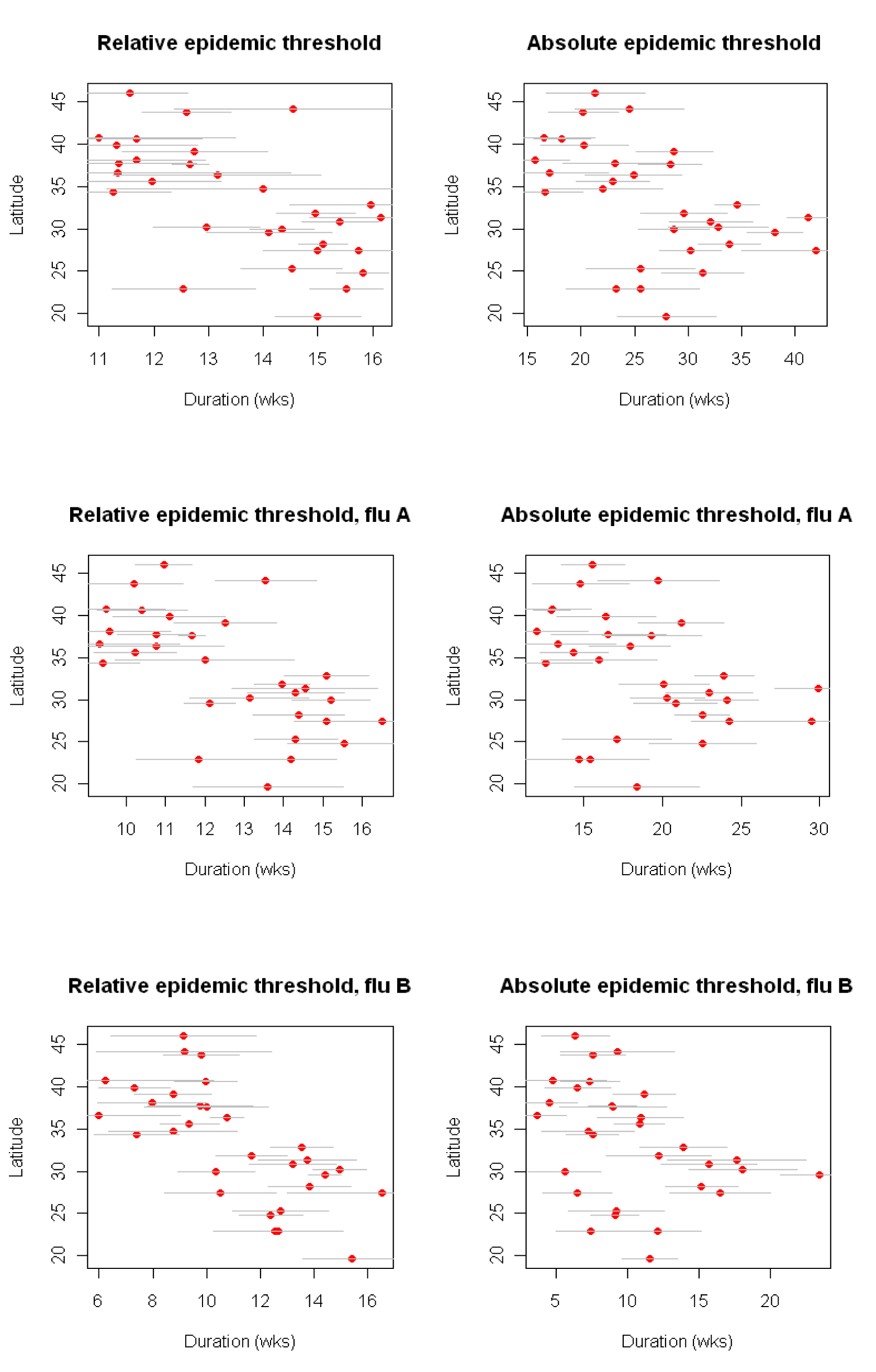

Supplement: Figure S6 — Latitudinal gradient in duration of influenza epidemics, by province and virus type. Duration is based on a relative measure (number of weeks with more than 2.5% of annual influenza virus isolated), or an absolute measure (number of weeks with more than 5% influenza percent positive). Top panels: influenza A and B combined; middle panels: influenza A; bottom panels: influenza B. Horizontal grey bars represent ±2 standard deviations based on inter-annual variability in the 7 study years. (TIF) [file pmed.1001552.s006.tif]

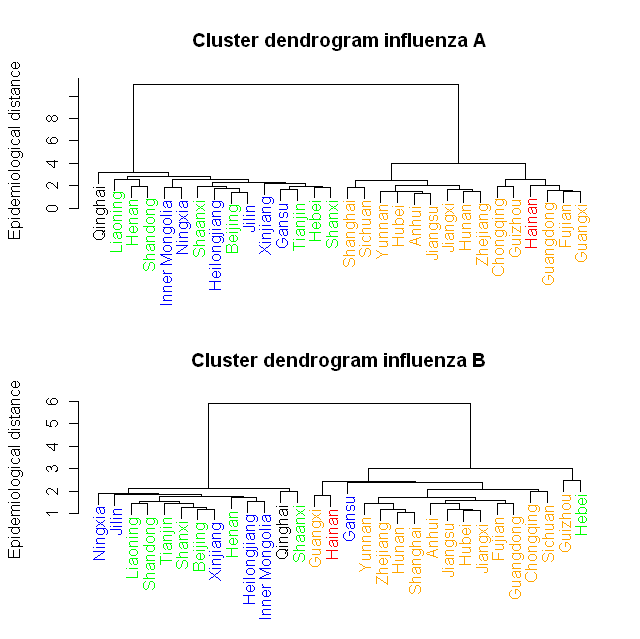

Supplement: Figure S7 — Influenza A (top) and B (bottom) epidemiological regions identified by cluster analysis. Epidemiological regions are based on hierarchical clustering (Ward's method), using the Euclidian distance between weekly standardized influenza time series. Provinces are color-coded by climatic region (black, cold-temperate; blue, mid-temperate; green, warm-temperate; orange, subtropical; red, tropical). (TIFF) [file pmed.1001552.s007.tif]

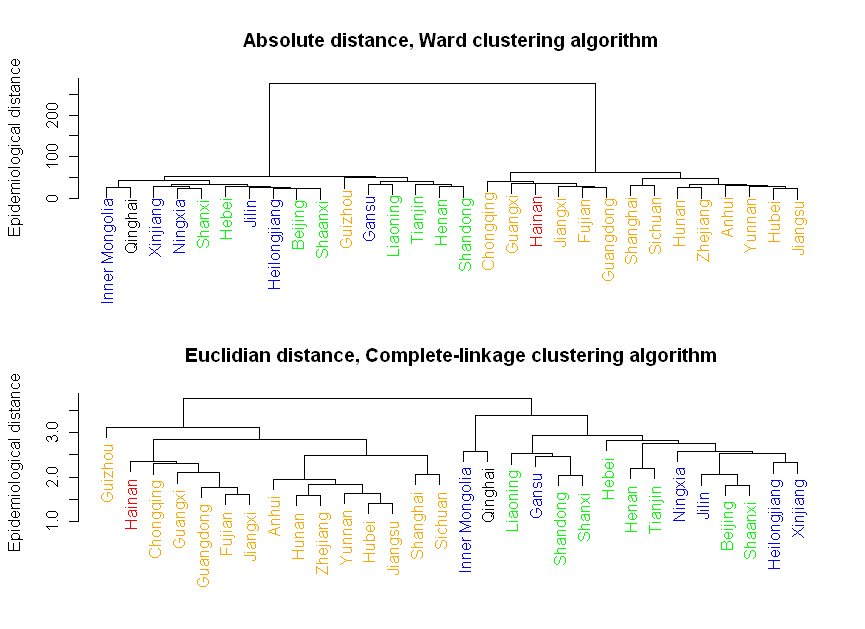

Supplement: Figure S8 — Sensitivity analyses on cluster algorithms used to define influenza epidemiological regions (compare with Figure 6 ). Top: using a different distance metric for pairwise differences between influenza time series (absolute distance, also known as Manhattan distance, instead of Euclidian distance). Bottom: using a different clustering algorithm (complete linkage instead of Ward). Analyses are based on total influenza activity. (TIFF) [file pmed.1001552.s008.tif]
