# Supplementary material for: Characterization of Regional Influenza Seasonality Patterns in China and Implications for Vaccination Strategies: Spatio-Temporal Modeling of Surveillance Data
Source: PLoS Med. 2013 Nov 19;10(11):e1001552. doi: 10.1371/journal.pmed.1001552 (PMC3864611; doi:10.1371/journal.pmed.1001552)
Supplement: Text S1 — Description of supplementary information. (DOC) [file pmed.1001552.s009.doc]

## Characterization of regional influenza seasonality patterns in China and implications for vaccination strategies:

## Supplementary Information

**Authors: Hongjie Yu^1^, Wladimir J Alonso^2^, Luzhao Feng^1^,** **Yi Tan^2^, Yuelong Shu^3^, Weizhong Yang^†1^, Cécile Viboud^†2^**

^1^ Division of Infectious Disease, Key Laboratory of Surveillance and Early-warning on Infectious Disease, Chinese Centre for Disease Control and Prevention, Beijing, China

^2^ Fogarty International Center, National Institutes of Health, Bethesda, MD, USA.

^3^National Institute for Viral Disease Control and Prevention, China CDC, Key Laboratory for Medical Virology, National Health and Family Planning Commission, Beijing, China

**1. Additional information regarding climatic data**

We included daily data for each climatological indicator and meteorological station with complete information for 2003-2011 (8 stations were excluded). Daily data were aggregated by month after removing unvalid values and/or outliers (defined here as the values above or below four standard deviations). City-level time series were aggregated by province by averaging their values. If one or more weeks had missing data at the province level, the values were obtained by linear interpolation of the remaining week values of the time series. Visual inspection of time series plots at all stages of this process was performed for consistency check.

To regress climate factors against estimated seasonal influenza parameters and avoid collinearity in climate indicators, we used the seasonal and annual minimums, maximums and means of each monthly climate factor. A similar procedure was used in a previous study linking influenza and climate on a global scale and indicated that annual climate thresholds were useful to define the geographic boundaries of influenza seasonality [1]

**2. Additional information regarding estimation of seasonal parameters**

a) Stage-1: Seasonal regression models (main analysis)

We used to following linear regression model to estimate the peak timing and amplitude of the annual and semi-annual periodicities of influenza activity in each province, as described in [2,3]:

flu_i_(t)=*a*+ *b_i_* *cos(2Pi*t/52.17) +  *c_i_* *sin(2Pi*t/52.17) +  *d_i_**cos(4Pi*t/52.17) +  *e_i_* *sin(4Pi*t/52.17) + ε(t)

where flu_i_(t) are the weekly standardized counts of influenza positive A (or B, or A+B combined) isolates in province *i*, where standardization is obtained by dividing weekly values by the annual number of specimens tested, *t* is a running index for week, and *a,b, c, d* and *e* are the intercept and seasonal terms to be estimated from the data. Equations for the seasonal parameters of interest are provided in the main text, including the amplitude of annual and semi-annual cycles, their ratio, and peak timing.

To obtain confidence intervals on seasonal parameter estimates, we used a block-bootstrap approach which is appropriate to take into account auto-correlation in time series data [4,5,6]. Inspection of the auto-correlation functions of the 30 province-specific flu_i_(t) time series suggested significant auto-correlation up to week 6, so that we elected to use a 6-week block bootstrap. We resampled 1,000 influenza time series from the 30 original flu_i_(t) province-level time series by bootstrap, estimated the corresponding seasonal coefficients based on the above model, and used the 2.5 and 97.5 percentiles of the bootstrapped distributions as confidence intervals. This procedure was done separately for influenza A, influenza B, and influenza A and B combined.

b) Stage-1 sensitivity analyses

We considered alternative seasonal regression models using a Poisson distribution and a log link (since we were using modified disease counts), with or without an offset for the number of annual specimens tested. Given that the two approaches gave sensibly similar results, we elected to use the linear model in the main analysis for the sake of simplicity.

We also conducted sensitivity analyses based on the percent influenza positive each week and using a logistic seasonal model with binomial errors, and obtained similar geographic gradients in estimated seasonal parameters as in the main model (Fig S5).

Finally, we fitted linear mixed regression models to jointly estimate seasonal characteristics in the 30 provinces, allowing for seasonality differences between broad regions (North/South China, considered as fixed effects), as well as province-specific random effects. This approach is particularly useful for sparse data as information can be borrowed from other provinces. We extracted the amplitude and peak timing of the annual and semi-annual periodicities based on the sum of fixed and random seasonal coefficients in each province and estimated the relative amplitude by dividing the absolute amplitude by the mean of each time series. Seasonal characteristics derived from the mixed model were extremely similar to those derived from the models stratified by province, indicating there was enough information in individual province-level time series. Inclusion of more refined grouping of Southern China (mid-latitude v. lower latitude Southern provinces, fixed effect) did not improve the fit of mixed models for any of the influenza subtypes. However, the seasonal coefficients (random effects) for the southernmost provinces were significantly different from the Southern group mean in these models (P<10^-9^), suggesting there is some statistical support for two influenza subregions within Southern China.

c) Stage-2 hierarchical Bayesian regression model of seasonal characteristics against environmental and population predictors

Because our seasonal characteristics (annual amplitude, peak timing, periodicity ratio, epidemic duration) were parameter estimates from stage-1 analysis rather than fixed quantities, we used Bayesian hierarchical regression models to identify putative predictors of seasonal patterns. In this approach, the estimation errors on the 30 province-specific seasonal parameters can be integrated as observation variances.

Assume that β_1i_ is a seasonal parameter of interest (eg, annual relative amplitude in province i), which we wish to predict on the basis of environmental and population factors. From stage-1 analysis, we obtained an estimate of β_1i_ , denoted here b_1i_.

We write:

b_1i_ ~ N(β_1i_, v_i_) , where v_i_ is the variance estimated from stage-1 by block bootstrap.

(Equ 1)

β_1i_ ~ N(α_0_ + α_1_*Predictor_1_+ α_2_*Predictor_2_+… α_j_*Predictor_j…_, τ^2^) (Equ 2)

where Predictors_j_ are putative seasonality predictors, α_j_ represent regression coefficients, and τ^2^ are regression errors.

We used Winbugs to estimate the αj and their errors, using non-informative initial priors

α_j_ ~ N(0, 1000)

τ^2^ ~ Uniform(0, 1000)

To facilitate estimation of a limited number of predictors in Winbugs, we first ran a stepwise multiple linear regression of seasonal estimates b_1i_ against all possible predictors, and used the identified subset of predictors in Equ 2.

**3. Supplementary Results**

*a) Model fit*

Figure S1 illustrates model fits for selected provinces representative of the 3 broad epidemiological regions observed in China. The fit of seasonal models was fair for all influenza subtypes combined (median R^2^=0.23, range 0.03-0.60), and for influenza A (median R^2^=0.25, range 0.03-0.50). Fit was weaker for influenza B (median R^2^=10%, range 0.1-26%). Model fit was not related to sampling intensity for any of the influenza outcomes (correlation between fit and no of specimens tested or no of influenza virus positive <0.20, P>0.19). Models for influenza A in high latitude provinces fit typically better than those in lower latitude regions (P<0.0001), while there was no geographical difference for influenza B (P=0.53). Overall, model fit was better for influenza A than B (paired Wilcoxon test, P<0.0001). Residuals showed some evidence of seasonality that was not accounted for in high latitude provinces; models also underestimated large influenza seasons, especially in the early study years (Fig S2).

*b) Seasonal parameters of aggregated influenza data (influenza A+B)*

We describe seasonal patterns separately for influenza A and B in the main text, and provide results for aggregated influenza A and B data below.

*Periodicity*. Estimates from time series models indicated strong annual periodicity in influenza activity in provinces above ~33^o^N latitude, and weaker annual periodicity at lower latitudes (Figure S1; median relative amplitude of annual cycle, 132% in the 15 Northern Temperate Provinces vs 28% in the 15 Southern provinces, Wilcoxon test, P<0.0001). Further, there was a strong latitudinal gradient in the amplitude of annual periodicity (Spearman rho=0.79, P<0.0001; Figure S2).

The relative importance of influenza semi–annual cycle also varied geographically, and 7 mid-latitude provinces ranging from 27.4^o^ - 31.3^o^N latitude experienced a dominant semi-annual cycle (ratio >0.5) (Figures 3 and 4). Overall, there was weak latitudinal gradient in importance of the semi-annual cycle, indicative of more intense semi-annual activity in the Southern half of China (Spearman rho=-0.44, P=0.01; Figure 4).

*Timing.* Estimates of influenza peak timing were concentrated in winter months in the 15 Northern temperate provinces (median phase Jan 12; range Dec 24- Feb 3; Figures 3 and 4). In contrast, epidemic timing was more variable in the 15 tropical and subtropical southern provinces. Mid-latitude provinces experienced semi-annual peaks in Jan-Feb and Jun-Aug, while the southernmost provinces had one major peak in spring (peak timing range, Apr-25 to Jun-15; Figure 3). There was a moderate latitudinal gradient in epidemic timing, indicative of increasingly later epidemics in the South (rho=-0.60, P<0.0005, Figure 4)

*Epidemic duration.* Average epidemic duration was longer in Southern than in Northern provinces based on an epidemic threshold set at a fraction of the annual number of cases (medians 15.1 vs 12.7 wks for a 2.5% threshold, Wilcoxon P=0.0003; Fig S7). The pattern of longer epidemic duration in the South was robust to using an alternative definition of epidemic duration based on the weekly proportion of respiratory specimens testing positive for influenza (medians 25.1 vs 19.0 wks for a 5% threshold, Wilcoxon P=0.0004). Estimates of epidemic duration were not affected by the surveillance scheme, with no difference between Northern provinces conducting surveillance during Oct-Mar and those with year-round sampling (difference<1.0 wk, P>0.24). Geographical differences in epidemic duration were robust to using more conservative thresholds to define of epidemic periods.

**References**

1. Tamerius J, Nelson MI, Zhou SZ, Viboud C, Miller MA, et al. (2011) Global influenza seasonality: reconciling patterns across temperate and tropical regions. Environ Health Perspect 119: 439-445.

2. Naumova EN, Jagai JS, Matyas B, DeMaria A, Jr., MacNeill IB, et al. (2007) Seasonality in six enterically transmitted diseases and ambient temperature. Epidemiol Infect 135: 281-292.

3. Alonso WJ, McCormick BJ (2012) EPIPOI: a user-friendly analytical tool for the extraction and visualization of temporal parameters from epidemiological time series. BMC Public Health 12: 982.

4. Weinberger DM, Simonsen L, Jordan R, Steiner C, Miller M, et al. (2012) Impact of the 2009 influenza pandemic on pneumococcal pneumonia hospitalizations in the United States. J Infect Dis 205: 458-465.

5. Alonso WJ, Viboud C, Simonsen L, Hirano EW, Daufenbach LZ, et al. (2007) Seasonality of influenza in Brazil: a traveling wave from the Amazon to the subtropics. Am J Epidemiol 165: 1434-1442.

6. Miller D (2004) Bootstrap 101: obtain robust confidence intervals for any statistic. <http://www2.sas.com/proceedings/sugi29/193-29.pdfAccessed> Montreal: SAS SUGI 29: 193–129.

**Figure S1:** **Fit of type-specific seasonal influenza models in 3 provinces representative of broad influenza epidemiological regions in China:** Shanxi (latitude 37.8^o^N, Northern temperate province experiencing winter seasonal influenza A and B epidemics), Hubei (latitude 30.9^o^N, mid-latitude subtropical province experiencing semi-annual influenza A epidemics), and Guangdong (22.9^o^N, Southern subtropical province experiencing late spring influenza A epidemics). Blue curve: observed cases standardized by the annual number of specimens tested; red curve: seasonal model. Grey lines mark Jan 1^st^ of each year, while the green line marks the 2009 A/H1N1 pandemic season, which was not included in the model fitting procedure. Model is based on a linear regression with harmonic terms for annual and semi-annual periodicities.

**Figure S2:** **Residuals of seasonal models presented in Fig S1 in 3 selected provinces.**

**Figure S3:** **Estimates of periodicity and timing of influenza epidemics in China (A and B combined).** Left) Timing of annual influenza peaks, in weeks. Timing is color coded by season. Center) Amplitude of annual periodicity, ranging from low (yellow) to high (red), as indicated in the legend. Amplitude is relative to the mean of the weekly influenza time series in each province. Right). Importance of semi-annual periodicities, measured by the ratio of the amplitude of the semi-annual periodicity to the sum of the amplitudes of annual and semi-annual periodicities. Yellow indicates strongly annual influenza epidemics, while red indicates marked semi-annual activity. See also Fig. S4

**Figure S4:** **Latitudinal gradients in seasonality of total influenza activity (A+B combined) in China.** Left: Relative amplitude of annual periodicity. Middle: Peak timing. Right: Contribution of the semi-annual cycle, measured by the ratio of the amplitude of the semi-annual cycle to the sum of the amplitudes of annual and semi-annual cycles. Open circles represent point estimates from seasonal regression models and horizontal dashed lines represent 95% confidence intervals based on 1,000 block-bootstrap samples. Purple lines represent linear regression of seasonal parameters against latitude (dashed line=unweighted regression, solid line=regression weighted by the inverse of the variance of province-specific seasonal estimates); R^2^ and P-values are indicated on the graphs. Colors represent different climatic zones (black: cold-temperate, blue mid-temperate, green warm-temperate, orange subtropical, red tropical).

**Figure S5:** **Sensitivity analysis on seasonal estimates for influenza A (top) and B (bottom) using a different model structure. Same as Figure 4 but using a logistic seasonal model with binomial errors to model weekly percent positive for influenza A and B (weekly no. of influenza positive / weekly no. of specimens tested).** Left: Relative amplitude of annual periodicity. Middle: Peak timing. Right: Contribution of the semi-annual cycle, measured by the ratio of the amplitude of the semi-annual cycle to the sum of the amplitudes of annual and semi-annual cycles. Open circles represent point estimates from seasonal regression models. Purple lines represent linear regression of seasonal parameters against latitude; P-values are indicated on the graphs. Colors represent different climatic zones (black: cold-temperate, blue mid-temperate, green warm-temperate, orange subtropical, red tropical).

**Figure S6: Latitudinal gradient in duration of influenza epidemics, by province and virus type.** Duration is based on a relative measure (no of weeks with more than 2.5% of annual influenza virus isolated), or an absolute measure (no of weeks with more than 5% influenza percent positive). Top panels: influenza A and B combined; middle panels: influenza A; bottom panels: influenza B. Horizontal grey bars represent +/- 2 standard deviations based on inter-annual variability in the 7 study years.

**Figure S7:** **Influenza A (top) and B (bottom) epidemiological regions identified by cluster analysis.** Epidemiological regions are based on hierarchical clustering (Ward’s method), using the Euclidian distance between weekly standardized influenza time series. Provinces are color-coded by climatic region (black: cold-temperate, blue: mid-temperate, green: warm temperate, orange: subtropical, red: tropical).

**Figure S8:** **Sensitivity analyses on the cluster analysis used to define influenza epidemiological regions (compare with Fig 5).** Top: using a different distance metric for pairwise differences between influenza time series (absolute distance, also known as Manhattan distance, instead of Euclidian distance). Bottom: using a different clustering algorithm (complete linkage instead of Ward). Analyses are based on total influenza activity.
